# Prevalence of malnutrition among hospital admissions to English NHS hospitals over two economically constrained decades: evidence from routine health data

**DOI:** 10.1136/bmjph-2024-002095

**Published:** 2025-07-17

**Authors:** Katharine Reeves, Samuel I Watson, Hannah Crothers, Paul Bird, Alexander Lawson, Magdalena Skrybant, Richard Lilford

**Affiliations:** 1 Research Development and Innovation, University Hospitals Birmingham NHS Foundation Trust, Birmingham, Birmingham, UK; 2 Department of Applied Health Sciences, University of Birmingham, Birmingham, Birmingham, UK; 3 Institute of Applied Health, University Hospitals Birmingham NHS Foundation Trust, Birmingham, Birmingham, UK; 4 Department of Clinical Chemistry, University Hospitals Birmingham NHS Foundation Trust, Birmingham, Birmingham, UK

**Keywords:** Public Health, statistics and numerical data, Body Mass Index

## Abstract

**Introduction:**

To examine the rates and distribution of hospital admissions for malnutrition in England between 2001 and 2021 using routine hospital admission data from Hospital Episode Statistics.

**Methods:**

A retrospective, longitudinal study using routinely collected data for the whole of England between 2001 and 2021 across private and National Health Service providers. We estimated age-standardised and age-specific rates of admission with diagnoses for protein-energy malnutrition or vitamin deficiencies among patients who did not suffer from a medical condition likely to cause malnutrition between 2001 and 2021. A geospatial analysis estimated the spatiotemporal distribution of age and income-adjusted risk.

**Results:**

Combining primary and secondary causes for admission, age-adjusted hospital admission rates doubled for protein-energy malnutrition (from 0.2/10 000 person years in 2001/2002 to 0.4/10 000 person years in 2020/2021). Vitamin deficiency admissions increased 5.4-fold from 0.5/10 000 person years in 2001/2002 to 2.6/10 000 person years in 2020/2021. However, these changes follow national recommendations to screen all inpatient admissions for malnutrition and a sharp increase in testing for vitamin deficiency. Changes in reporting are therefore likely to be largely (or completely) an artefact of awareness and testing. Furthermore, there was no increase in cases of protein-energy or vitamin deficiency when malnutrition was the primary cause of admission. Overall, poorer areas did have higher malnutrition admission rates than richer areas.

**Conclusions:**

There was a steep rise in admissions with a code for protein-energy malnutrition and for vitamin deficiency in any diagnosis position among adults. However, increased diagnosis could be explained by new guidelines and increased testing. Moreover, there was no increase in admission with either type of malnutrition when this was the primary cause of admission. The pattern was similar for all admissions and admissions excluding alcohol-related conditions.

**WHAT IS ALREADY KNOWN ON THIS TOPIC**

- A large increase in the number of food banks and reports of food insecurity may suggest an increased risk of malnutrition in England.

**WHAT THIS STUDY ADDS**

- Our analysis showed a rise in hospital admissions with a diagnosis code for protein-energy malnutrition and for vitamin deficiency among adults. We showed that the larger increase for vitamin deficiency was likely an artefact of increased testing.

**HOW THIS STUDY MIGHT AFFECT RESEARCH, PRACTICE OR POLICY**

- Our findings underscored the difficulty in making causal assumptions about a high-income nation’s underlying nutritional status from hospital admission data.

## Introduction

 There have been large social and economic changes in England in the last 20 years, with the National Health Service (NHS) and social care services experiencing large increases in funding between 2001 and 2010, followed by a policy of ‘austerity’ in the following decade. Funding for social care spending, for example, declined by 31% per person in the years 2009–2010 to 2017–2018.^1^ The period of ‘austerity’ was accompanied by a proliferation of ‘food banks’, community-led organisations that provide food at no cost to those in need, supported by the non-governmental sector. There are now over 2200 food banks in the UK as a whole; 1300 are run by the charity Trussell Trust.^2^ Nevertheless, there are many reports of food insecurity and of hunger, with one estimate placing the prevalence of ‘severe food insecurity’ at 3% in the UK.^3^ In 2013, the Institute for Fiscal Studies reported a decrease in the number of calories purchased and substitution to foods with poor nutritional value with suggestions of an emerging “public health emergency”.^4^ A 2018 report suggested the UK was doing poorly in its progress towards Sustainable Development Goal 2 (zero hunger) with few policies in place to tackle food insecurity, which was estimated to affect 2.2 million households.^5^ Taken together, the evidence may suggest an increased risk of malnutrition in England. The aim of our study, therefore, is to track hospital admission with malnutrition diagnoses likely to be attributable to food insecurity resulting from the above adverse economic circumstances.

In this study, we used NHS hospital admissions data to estimate malnutrition rates and the distribution of cases of malnutrition in England over the period 2001 to 2021, excluding medical diseases characteristically associated with malnutrition.

There are numerous studies of malnutrition in the community based on population surveys of different types.^6^ As stated, there are reports of rising use of foodbanks (Trussell Trust), but these could reflect supply rather than demand. There are also very large surveys of malnutrition among hospital inpatients,^7 8^ but these do not track rates over long periods and do not estimate community-acquired malnutrition by excluding patients admitted to hospital with diseases classically associated with malnutrition, such as cancer, AIDS, tuberculosis or malabsorption.

## Materials and methods

### Study design and setting

This paper follows the REporting of studies Conducted using Observational Routinely collected Data guidelines for studies conducted using routinely-collected healthcare data (see online supplemental material 1).^9^

We conducted a retrospective, observational, longitudinal study using routinely collected healthcare data to estimate rates and distribution of admissions to NHS hospitals with a diagnosis of protein-energy malnutrition or vitamin deficiency in England (excluding vitamin D), between 1 April 2001 and 31 March 2021.

### Data extraction

The International Classification of Diseases (ICD)-10 codes used to identify diagnoses of malnutrition are shown in online supplemental material 2, split into protein-energy malnutrition (‘malnutrition’) and vitamin deficiency (‘vitamin deficiency’). We analysed the Hospital Episode Statistics (HES) data at the level of spells of continuous care by a single provider institution and extracted all spells with one of these malnutrition diagnosis codes in any diagnosis position (primary or secondary). Only the first spell for each patient was included. We excluded cases where the malnutrition code was accompanied by a medical diagnosis that may directly cause malnutrition, including cancer, HIV, malabsorption syndrome and tuberculosis (complete list in online supplemental material 2). We examined for protein-energy and vitamin deficiency, excluding vitamin D, because we were aware that increasing public and medical awareness of vitamin D would have led to increased testing. We also examined the effect of excluding diagnoses related to alcohol consumption in order to isolate the specific effect of diet on an increase in malnutrition secondary to any increase in alcoholism (annual figures shown in online supplemental material 3). Participants aged under 1 year, over 120 years or with missing age information were excluded. The Lower Super Output Area 2011 (LSOA11) code in the HES record was used to aggregate data to MSOAs. The spatiotemporal analysis covered the period 2010/2011–2020/2021 so that LSOAs would be consistent over the entire period.

### Additional study

When we came to analyse the data regarding admissions associated with vitamin deficiency, we realised that a rise in cases over time could be an artefact of increased testing. A recent study in the Netherlands set out to investigate the reasons for increased testing not only for vitamin D (which we exclude) but also vitamin B_12_. They found that this was often due to conflicting information about the usefulness of vitamin tests, different motivations of General Practitioners (GPs) and patients, and GPs’ tendency to avoid conflict and reassure patients by ordering tests.^10^ We, therefore, decided to investigate for any change in testing rates and the proportion of positive tests. By a very large margin, the vitamin that was most commonly recorded as a cause for admissions was vitamin B_12_ (automatically accompanied by a test for folic acid). We, therefore, tracked testing for vitamin B_12_ at the Queen Elizabeth Hospital Birmingham (QEHB). Investigations at QEHB are recorded on the Birmingham Systems Prescribing Information and Communications System. The normal range for B_12_ is 187–883 ng/L. Therefore, a result was classified as deficient when the result was below the normal range. We tried to obtain these data from other hospital services, but the information was recorded in so many different ways across chemical pathology that it proved infeasible to aggregate.

### Statistical analysis

We described rates of admission for malnutrition in the HES admitted patient care database which contains all inpatient and day case admissions funded by the NHS in England. National trends were examined over the period 1 April 2001–31 March 2021.

We calculated rates of admission for malnutrition per 10 000 population per year using population estimates by year and age from the Office of National Statistics (ONS).^5^ Age-standardised rates were calculated by splitting admissions into the age categories 1–17 years, 18–24 years, 25–34 years, 35–44 years, 45–54 years, 55–64 years, 65–74 years, 75–84 years and 85+years, using the 2020 population as the standard population.

We then used the same data, aggregated to Middle Layer Super Output Areas (MSOAs), to conduct a geospatial statistical analysis to model the spatial and temporal variation of malnutrition across England between 2010/2011 and 2020/2021. The largest changes in incidence were observed over this period, and during this period, census-level areas were constant, having changed with the census in 2011. Admission counts were aggregated to MSOAs, of which there are 6791 in England. We specified a Bayesian Log Gaussian Cox Process model that ‘smooths’ rates over space and time and which allows for incidence data aggregated to irregular areas.^11 12^ Given the size of the dataset, we used an accurate approximation to the Gaussian process to facilitate computation,^13^ using the R package rts2. We included in the model a population offset (using ONS predictions of MSOA populations for each year) and indicators for year, proportion of the population in each age category above, and indicators for quintile of income net of housing costs. We predicted incidence rate ratios for 2020/2021 vs 2010/2011 and age and income-adjusted smoothed relative risks for 2020/2021.

### Data access and cleaning methods

Investigators at University Hospitals Birmingham (UHB) had access to pseudonymised, patient-level HES data for the purposes of this study. Data were cleaned using the inclusion criteria listed above and aggregated for reporting purposes. This observational study was registered with the local Clinical Audit Department (Clinical Audit Registration and Management System number 15147). Data were used in line with the data sharing agreement with NHS Digital.

### Patient involvement

No patients were involved in setting the research question or the outcome measures, nor were they involved in developing plans for recruitment, design or implementation of the study. No patients were asked to advise on interpretation or writing up of results. There are no plans to disseminate the results of the research to study participants or the relevant patient community.

### Ethics approval

Ethics approval was not needed for this study, and this was confirmed by the University of Birmingham Research Ethics team.

## Results

### Protein-energy malnutrition and vitamin deficiency

Between 1 April 2001 and 31 March 2021, there were 30 705 admissions (in any diagnostic position (primary or secondary)) for protein-energy malnutrition and 143 849 for vitamin deficiency across 261 private and 342 NHS providers. As stated, these were for diagnoses after excluding vitamin D and admissions for malnutrition-causing disease and in any diagnostic position (ie, primary and secondary diagnoses). The findings are shown in online supplemental material 4.

### Trends by diagnosis type

**Figure 1:**
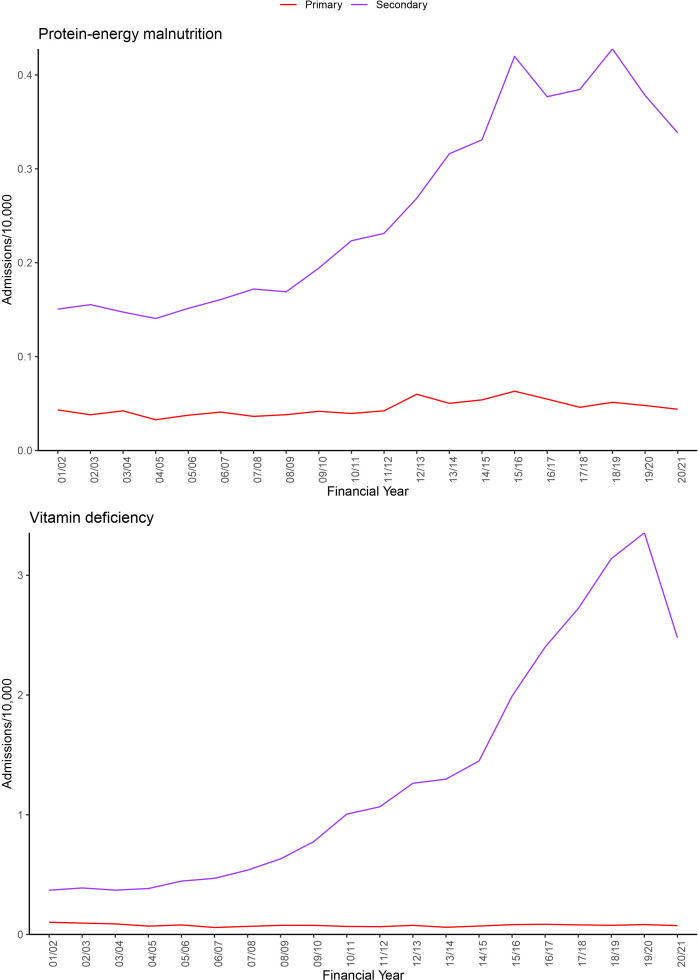
Admissions with protein-energy malnutrition or vitamin deficiency by diagnosis position. Admission rates are age-standardised to the 2020 population. Note that a small number of admissions contained a diagnosis for both types of malnutrition and hence will be included in both groups.

More admissions contained a diagnosis for vitamin deficiency than protein-energy malnutrition. Figure 1 shows trends for admissions with protein-energy malnutrition or vitamin deficiency by any diagnosis position and in the primary position.

The age-adjusted rate for admissions containing a diagnosis for protein-energy malnutrition in any position doubled, increasing from 0.2/10 000 person years in 2001/2002 to 0.4/10 000 person years in 2020/2021. The age-adjusted rate for admissions containing a diagnosis for vitamin deficiency in any position increased 5.4-fold from 0.5/10 000 person years in 2001/2002 to 2.6/10 000 person years in 2020/2021 (online supplemental material 5). It was hard to identify clear inflection points in the graphs in figure 1 other than to say the increase seemed to occur from the mid-2000s to the mid-2010s. The number of spells with an alcohol-related diagnosis was relatively low (online supplemental table 3), and excluding those cases made little difference to the rates of protein-energy or vitamin deficiency admissions. While there were stark increases in cases of protein-energy or vitamin deficiency in any (ie, primary or secondary) position, there was no sign of an increase in these diagnoses in the primary position (figure 1).

### Trends by age group

**Figure 2:**
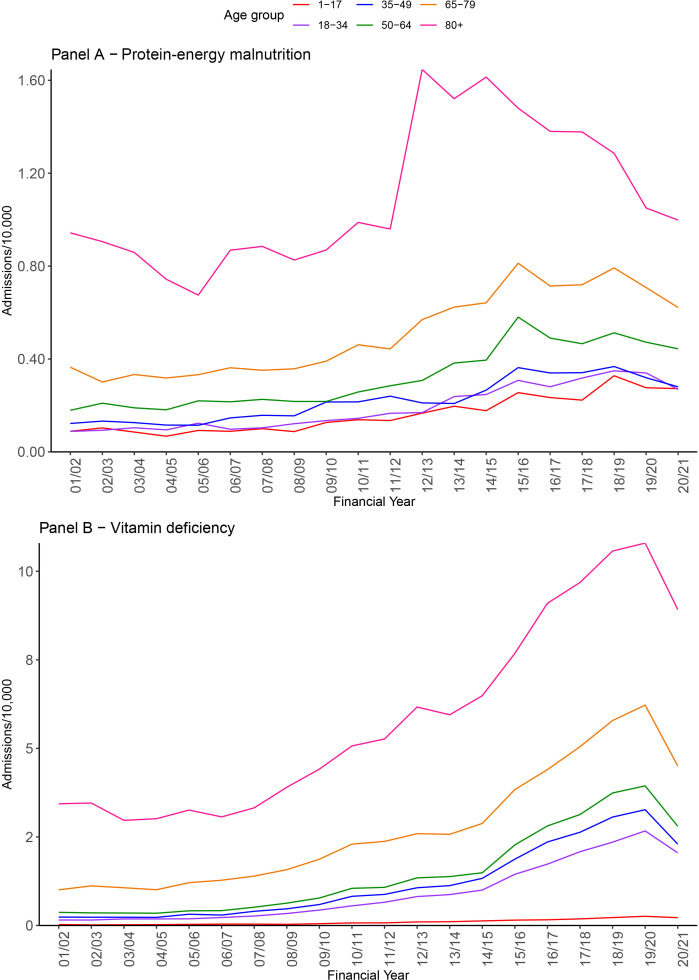
Admissions with protein-energy malnutrition or vitamin deficiency in any diagnosis position by age group.

We provide a breakdown for protein-energy malnutrition and vitamin deficiency admissions by age group in figure 2. Admission rates were highest in the older age groups, but a similar proportional increase in admissions was seen in all age groups. There was a sharp rise in protein-energy deficiency diagnoses specifically in the older age group in 2012/2013. There was a dip for all in the year 2019/2020 due to the COVID-19 pandemic.

### Type of vitamin deficiency

Table 1 shows the most common ICD-10 codes for vitamin deficiency diagnoses in a primary or secondary position. ‘Deficiency of other specified B group vitamins’ included biotin, folate, folic acid, pantothenic acid but vitamin B_12_ made much the largest contribution to the increase in this category.

**Table 1:** Most frequent ICD-10 codes for diagnoses of vitamin deficiency in any position

ICD10	N	%
E538 - deficiency of other specified B group vitamins	141 998	98.27%
E639 - nutritional deficiency, unspecified	1181	0.82%
E54X - ascorbic acid deficiency	640	0.44%
D520 - dietary folate deficiency anaemia	534	0.37%

ICD, International Classification of Diseases.

### B vitamin testing

**Figure 3:**
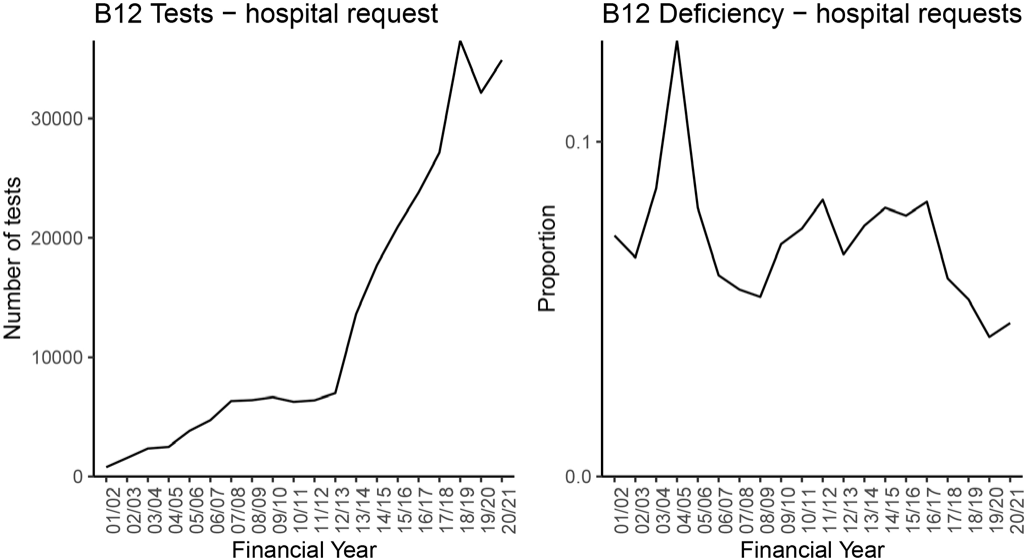
The number of patients tested for B_12_ deficiency at Queen Elizabeth Hospital Birmingham from 2001/2002 to 2020/2021 and the proportion of patients with a deficient result.

The plots in figure 3 show the annual rate of testing and the proportion with a deficient result for vitamin B_12_ at QEHB. Vitamin B_12_ made up a large majority of B vitamin test requests, so we focused on this for our analysis. The rate increased over time, but the proportion of these tests with a vitamin deficiency result did not show an increase over time.

### Geospatial analysis

Table 2 reports the estimated incidence rate ratios by MSOA income quintile in England averaged over the time period. For both types of malnutrition outcome, the age-adjusted rates showed a strong association with income levels; the highest income quintiles had rates on average 36% (95% CI: 38 to 34%) and 40% (45% to 36%) lower than the lowest income quintiles for vitamin deficiencies and protein-energy malnutrition, respectively.

**Table 2:** Estimated incidence rate ratios (95% CI) for admission to hospital with vitamin deficiencies and protein-energy malnutrition for quintiles of income net of housing costs for MSOAs in England

Income quintile	Vitamin deficiencies	Protein-energy malnutrition
1–Lowest	1–Reference	1–Reference
2	0.94 (0.92 to 0.97)	0.83 (0.79 to 0.88)
3	0.87 (0.85 to 0.89)	0.76 (0.63 to 0.81)
4	0.76 (0.74 to 0.79)	0.68 (0.63 to 0.72)
5–Highest	0.64 (0.62 to 0.66)	0.60 (0.55 to 0.64)

Online supplemental material 6 shows the age and income-adjusted smoothed relative risk for 2020/2021. This showed spatial variation that could not be explained by differences in age or economic status across areas of the country. The area served by University Hospitals of Morecambe Bay NHS Foundation Trust showed the highest adjusted relative risk for vitamin deficiencies, up to six times higher than the national age and income-adjusted average rate. We investigated this and discovered it was due to a subsequently corrected laboratory calibration error. Other areas with high relative risk of vitamin deficiency included Gloucester, Manchester and Liverpool. For protein-energy malnutrition, Liverpool and Merseyside, particularly around Arrowe Park Hospital, had a 10 times higher age and income-adjusted relative risk than the average for England.

Online supplemental material 7 shows change over time (2010/2011 vs 2020/2021) in prevalence of malnutrition. The figure shows an increase over time that was much more variable geographically for vitamin deficiency than for protein-energy malnutrition. Thus, the steep rise in vitamin deficiency recorded in figures1  2 was driven by much greater levels of recorded malnutrition in some areas than in others.

## Discussion

### Key results

There was a substantial increase in admissions for malnutrition over the last 20 years, with the increase starting in the mid-2000s. The increase applied to both protein-energy and vitamin malnutrition, but the magnitude of the increase was much greater in the latter case. The increase applied to both children and adults. There was also an increase in alcohol-related admissions (as shown in online supplemental material 3); however, the increase was not driven by alcohol which was given as a reason for admission in only a minority of malnutrition admissions.

The changes observed here could have three non-exclusive explanations: (1) a causal effect of rising poverty causing food insecurity (and hence inadequate diet); (2) an artefact of testing and increased awareness or (3) change in identification of diseases not captured in our exclusion criteria. It is impossible to resolve this issue completely, but evidence for or against these different explanations can be found in the data and from external sources.

### Testing and awareness

Regarding vitamins, we think increased testing was the likely cause; we found that testing rates increased with a similar pattern to admissions at QEHB and there was national (and indeed international) concern over increased, and possibly over-testing. A further clue was provided by the proportion of those tested who had a positive result. This proportion shows no increase and a possible decrease. Many areas of increased age-adjusted and income-adjusted risk also adhered to NHS Trust boundaries, suggesting that it was differing testing behaviour, rather than underlying disease, that drove changes in vitamin deficiency. It would seem that most, if not all, of the increase in admissions was an artefact of increased testing. A report was published by the Guardian newspaper, which also found increased admissions with vitamin deficiency, arguing that this was caused by poverty. However, the report did not provide any data on testing behaviour.^14^ Note also that there was no increase in vitamin deficiency as the *primary* reason for admission (figure 1).

Protein-energy malnutrition is a clinical diagnosis. The doubling in prevalence across all age groups could be the result of increased recognition of the problem and/or a change in coding behaviour. We knew of no incentive to increase coding; a problem that occurs when remuneration is influenced by procedure or diagnostic rates.^15^ It is more likely that diagnostic thresholds had decreased due to general awareness of malnutrition, especially as the National Institute for Health and Care Excellence issued guidance in 2006 recommending that all patients should be screened for malnutrition on admission. This is supported by the shape of the graphs in figure 1, which show an increasing trend after 2006. Admissions where protein-energy malnutrition was the *primary* diagnosis had changed little and certainly did not mirror the more dramatic changes seen with respect to protein-energy malnutrition as a secondary diagnosis. This may be taken as evidence that there was no underlying increase in malnutrition due to poverty. The argument here would be that poverty-related malnutrition would affect prevalence in both the primary and secondary positions.

### What, if not an artefact, is the causal process behind the increase in admissions?

When we conceived this study, we hypothesised that malnutrition would increase as a result of the financial crisis in 2008. Reference to the trend lines in this study supported our hypothesis as there was a clear acceleration in malnutrition in 2008. The recession ended in the last quarter of 2009 but malnutrition admissions continued to increase. The financial crisis triggered a period of fiscal contraction known as austerity, and this provided a plausible underlying cause of the increase in malnutrition admissions. This trend continued over the period around the UK’s exit from the European Union and into the COVID era, during which malnutrition admissions mirrored the period when admissions as a whole declined. While these political and macro-economic changes may represent the ultimate determinants of malnutrition, our study was silent on the causal chain through which these distal determinants ultimately impacted on individual lives and numerous policies, such as withdrawal of Sure Start or introduction of food banks.

### The role of food banks

Food banks have been in place in the UK for around 20 years. The Trussell Trust opened its first food bank in 2000 with numbers steadily increasing to 1300 today. Food parcels given out increased most rapidly between 2012 and 2016. This followed a precipitous rise in admissions, and it is possible that the rise would have been even steeper but continued at a similar pace, but for the food banks. We should also consider the alternative possibility that food banks stimulated referral to hospitals, providing a type of supply-induced demand. This idea was not supported by the observation that malnutrition was a secondary diagnosis in the great majority of admissions with malnutrition.

### Limitations

As with most observational studies, we can find associations but cannot unravel the underlying mechanisms and, as with most database studies, we must rely on reasonably accurate coding. Moreover, our search is unlikely to exclude all possible medical causes of malnutrition that can occur across a wide range of chronic diseases of the lung, liver and nervous system. If the underlying prevalence of such conditions increased in the population, then that would show up in our data. For example, the incidence of vitamin B_12_ deficiency may increase in parallel with bariatric and other gastric surgeries. However, this idea is not supported by our finding that the positivity rate among those tested tended to fall.

Within the methodological paradigm of a database study, we thought the greatest limitation in our data lay in their origin in but one country. It will be most interesting to compare across countries taking into account different policy responses to similar levels of economic challenge. Likewise, the effect of non-governmental interventions, such as food banks, warrants further investigation.

Our analysis revealed something of the difficulty of linking economic circumstances to health effects of under-nutrition in a high-income country. Food purchasing behaviour (for example, from supermarket data) likely provides a better measure of over-nutrition than under-nutrition. As we have shown, examining health outcomes is fraught with difficulty. Population surveys, if large enough, might provide the best evidence in this area. Malnutrition is a complex medical entity with multiple overlapping causes, including recognition, testing behaviour, uptake of surgery, food scarcity and a very wide range of medical diseases, among which it is under-recognised. Our study shows that, at least in high-income countries, it is difficult to measure its true prevalence and determine its causes.

### Interpretation

We are left very uncertain regarding causal inference. The main or only cause of increased vitamin admissions was likely due to testing behaviour. Protein-energy malnutrition may have been due to a causal relation with poverty and/or an artefact of awareness and change in diagnostic thresholds. Had malnutrition increased as a primary diagnosis, we would have been on stronger ground in arguing for a causal explanation. We thus concluded that national admissions data could not be used in a high-income country to make causal implications about a nation’s underlying nutritional status.

## Supplementary material

10.1136/bmjph-2024-002095 online supplemental file 1

## Data Availability

Data may be obtained from a third party and are not publicly available.

## References

[1] Crawford R, Stoye G, Zaranko B (2021). Long-term care spending and hospital use among the older population in England. J Health Econ.

[2] Francis-Devine B (2024). Food banks in the uk.

[3] Pool U, Dooris M (2022). Prevalence of food security in the UK measured by the Food Insecurity Experience Scale. J Public Health (Oxf).

[4] U.S. Department of Agriculture (2023). Food security in the U.S. https://www.ers.usda.gov/topics/food-nutrition-assistance/food-security-in-the-u-s/.

[5] Office for National Statistics (2023). Population estimates for england and wales: mid-2022. https://www.ons.gov.uk/peoplepopulationandcommunity/populationandmigration/populationestimates/bulletins/populationestimatesforenglandandwales/mid2022.

[6] Stratton R, Cawood A, Anderson L (2023). Malnutrition and nutritional care survey in adults.

[7] Edington J, Boorman J, Durrant ER (2000). Prevalence of malnutrition on admission to four hospitals in England. Clin Nutr.

[8] Russell CA, Elia M (2010). Malnutrition in the UK: where does it begin?. Proc Nutr Soc.

[9] REporting of studies Conducted using Observational Routinely-collected Data (2019). What is record?. https://www.record-statement.org/.

[10] Hofstede H, van der Burg HAM, Mulder BC (2019). Reducing unnecessary vitamin testing in general practice: barriers and facilitators according to general practitioners and patients. BMJ Open.

[11] Møller J, Syversveen AR, Waagepetersen RP (1998). Log Gaussian Cox Processes. Scandinavian J Statistics.

[12] Li Y, Brown P, Gesink DC (2012). Log Gaussian Cox processes and spatially aggregated disease incidence data. Stat Methods Med Res.

[13] Solin A, Särkkä S (2020). Hilbert space methods for reduced-rank Gaussian process regression. Stat Comput.

[14] Devlin H (2023). Surge in number of people in hospital with nutrient deficiencies, NHS figures show.

[15] Bien Z, Fowler AJ, Robbins AJ (2022). Trends in Hospital Admissions Associated with an Acute Kidney Injury in England 1998–2020: a Repeated Cross-Sectional Study. SN Compr Clin Med.

