# Supplementary material for: Prevalence of malnutrition among hospital admissions to English NHS hospitals over two economically constrained decades: evidence from routine health data
Source: BMJ Public Health. 2025 Jul 17;3(2):e002095. doi: 10.1136/bmjph-2024-002095 (PMC12273158; doi:10.1136/bmjph-2024-002095)

Supplementary Materials

1. The RECORD statement
2. Table: ICD10 Codes
3. Table: Descriptive characteristics
4. Table: Admissions for malnutrition with an alcohol-related diagnosis
5. Figure: Selection of admissions with dietary related malnutrition
6. Figure: Relative risk by middle-layer super output area in England – 2020/21
7. Figure: Predicted incidence rate ratio for incidence in 2020/21 versus 2010/11 by middle-layer super output area in England

Supplementary Material 1

The RECORD statement – checklist of items, extended from the STROBE statement, that should be reported in observational studies using routinely collected health data

	Item No.	STROBE items	Location in manuscript where items are reported	RECORD items	Location in manuscript where items are reported
**Title and abstract**					
	1	(a) Indicate the study's design with a commonly used term in the title or the abstract (b) Provide in the abstract an informative and balanced summary of what was done and what was found		RECORD 1.1: The type of data used should be specified in the title or abstract. When possible, the name of the databases used should be included.<br><br>RECORD 1.2: If applicable, the geographic region and timeframe within which the study took place should be reported in the title or abstract.<br><br>RECORD 1.3: If linkage between databases was conducted for the study, this should be clearly stated in the title or abstract.	Abstract<br>Page 2<br><br>Title + Abstract<br>Page 2<br><br>N/a

**Introduction**					
Background rationale	2	Explain the scientific background and rationale for the investigation being reported			Introduction<br><br>Page 4
Objectives	3	State specific objectives, including any prespecified hypotheses			Introduction<br><br>Page 4
**Methods**					
Study Design	4	Present key elements of study design early in the paper			Study design and setting<br><br>Page 5
Setting	5	Describe the setting, locations, and relevant dates, including periods of recruitment, exposure, follow-up, and data collection			Study design and setting<br><br>Page 5
Participants	6	*(a) Cohort study* - Give the eligibility criteria, and the sources and methods of selection of participants. Describe methods of follow-up<br><br>*Case-control study* - Give the eligibility criteria, and the sources and methods of case ascertainment and control selection. Give the rationale for the choice of cases and controls		RECORD 6.1: The methods of study population selection (such as codes or algorithms used to identify subjects) should be listed in detail. If this is not possible, an explanation should be provided.<br><br>RECORD 6.2: Any validation studies of the codes or algorithms used to select the population should be referenced. If validation was conducted for this study	Data extraction + Supplementary Material 2<br><br>Pages 5-6<br><br>N/a

		Cross-sectional study - Give the eligibility criteria, and the sources and methods of selection of participants<br><br>*(b) Cohort study* - For matched studies, give matching criteria and number of exposed and unexposed<br><br>*Case-control study* - For matched studies, give matching criteria and the number of controls per case		and not published elsewhere, detailed methods and results should be provided.<br><br>RECORD 6.3: If the study involved linkage of databases, consider use of a flow diagram or other graphical display to demonstrate the data linkage process, including the number of individuals with linked data at each stage.	N/a
Variables	7	Clearly define all outcomes, exposures, predictors, potential confounders, and effect modifiers. Give diagnostic criteria, if applicable.		RECORD 7.1: A complete list of codes and algorithms used to classify exposures, outcomes, confounders, and effect modifiers should be provided. If these cannot be reported, an explanation should be provided.	Statistical analysis<br><br>Page 7
Data sources/ measurement	8	For each variable of interest, give sources of data and details of methods of assessment (measurement).<br><br>Describe comparability of assessment methods if there is more than one group			Data extraction<br><br>Pages 5-6
Bias	9	Describe any efforts to address potential sources of bias			N/a

Study size	10	Explain how the study size was arrived at			Data extraction<br><br>Pages 5-6
Quantitative variables	11	Explain how quantitative variables were handled in the analyses. If applicable, describe which groupings were chosen, and why			Data extraction<br><br>Pages 5-6
Statistical methods	12	(a) Describe all statistical methods, including those used to control for confounding<br><br>(b) Describe any methods used to examine subgroups and interactions<br><br>(c) Explain how missing data were addressed<br><br>(d) *Cohort study* - If applicable, explain how loss to follow-up was addressed<br><br>*Case-control study* - If applicable, explain how matching of cases and controls was addressed<br><br>*Cross-sectional study* - If applicable, describe analytical methods taking account of sampling strategy			Statistical analysis<br><br>Page 7

		(e) Describe any sensitivity analyses			
Data access and cleaning methods		..		RECORD 12.1: Authors should describe the extent to which the investigators had access to the database population used to create the study population.<br><br>RECORD 12.2: Authors should provide information on the data cleaning methods used in the study.	Data access and cleaning methods<br><br>Page 8
Linkage		..		RECORD 12.3: State whether the study included person-level, institutional-level, or other data linkage across two or more databases. The methods of linkage and methods of linkage quality evaluation should be provided.	N/a
**Results**					
Participants	13	(a) Report the numbers of individuals at each stage of the study (*e.g.*, numbers potentially eligible, examined for eligibility, confirmed eligible, included in the study, completing follow-up, and analysed)<br><br>(b) Give reasons for non-participation at each stage.		RECORD 13.1: Describe in detail the selection of the persons included in the study (*i.e.,* study population selection) including filtering based on data quality, data availability and linkage. The selection of included persons can be described in the text and/or by means of the study flow diagram.	Supplementary Material 5

		(c) Consider use of a flow diagram			
Descriptive data	14	(a) Give characteristics of study participants (*e.g.*, demographic, clinical, social) and information on exposures and potential confounders<br><br>(b) Indicate the number of participants with missing data for each variable of interest<br><br>(c) *Cohort study* - summarise follow-up time (*e.g.*, average and total amount)			Supplementary Material 3
Outcome data	15	*Cohort study* - Report numbers of outcome events or summary measures over time<br><br>*Case-control study* - Report numbers in each exposure category, or summary measures of exposure<br><br>*Cross-sectional study* - Report numbers of outcome events or summary measures			Supplementary Material 5
Main results	16	(a) Give unadjusted estimates and, if applicable, confounder-adjusted estimates and their precision (e.g., 95% confidence interval). Make clear which			Results<br><br>Pages 8-11

		confounders were adjusted for and why they were included<br><br>(b) Report category boundaries when continuous variables were categorized<br><br>(c) If relevant, consider translating estimates of relative risk into absolute risk for a meaningful time period			
Other analyses	17	Report other analyses done—e.g., analyses of subgroups and interactions, and sensitivity analyses			Results<br><br>Pages 11-13
**Discussion**					
Key results	18	Summarise key results with reference to study objectives			Key Results<br><br>Page 13
Limitations	19	Discuss limitations of the study, taking into account sources of potential bias or imprecision. Discuss both direction and magnitude of any potential bias		RECORD 19.1: Discuss the implications of using data that were not created or collected to answer the specific research question(s). Include discussion of misclassification bias, unmeasured confounding, missing data, and changing eligibility over time, as they pertain to the study being reported.	Limitations<br><br>Pages 15-16
Interpretation	20	Give a cautious overall interpretation of results considering objectives, limitations, multiplicity of			Interpretation<br><br>Page 16

		analyses, results from similar studies, and other relevant evidence			
Generalisability	21	Discuss the generalisability (external validity) of the study results			N/a
**Other Information**					
Funding	22	Give the source of funding and the role of the funders for the present study and, if applicable, for the original study on which the present article is based			Funding<br><br>Page 17
Accessibility of protocol, raw data, and programming code		..		RECORD 22.1: Authors should provide information on how to access any supplemental information such as the study protocol, raw data, or programming code.	Accessibility of protocol, raw data and programming code<br><br>Page 17

*Reference: Benchimol EI, Smeeth L, Guttmann A, Harron K, Moher D, Petersen I, Sørensen HT, von Elm E, Langan SM, the RECORD Working Committee. The REporting of studies Conducted using Observational Routinely-collected health Data (RECORD) Statement. *PLoS Medicine* 2015; in press.

*Checklist is protected under Creative Commons Attribution (CC BY) license.

Supplementary Material 2

Table: ICD10 codes

ICD10 Code	Description	Category
E40X	E40X - Kwashiorkor	Malnutrition
E41X	E41X - Nutritional marasmus	Malnutrition
E42X	E42X - Marasmic kwashiorkor	Malnutrition
E43X	E43X - Unspecified severe protein-energy malnutrition	Malnutrition
E440	E440 - Moderate protein-energy malnutrition	Malnutrition
E441	E441 - Mild protein-energy malnutrition	Malnutrition
E45X	E45X - Retarded development following protein-energy malnutrition	Malnutrition
E46X	E46X - Unspecified protein-energy malnutrition	Malnutrition
E880	E880 - Disorders of plasma-protein metabolism, not elsewhere classified	Malnutrition
R636	R636 - Insufficient intake of food and water due to self neglect	Malnutrition
T730	T730 - Effects of hunger	Malnutrition
D520	D520 - Dietary folate deficiency anaemia	Vitamin deficiency
E500	E500 - Vitamin A deficiency with conjunctival xerosis	Vitamin deficiency
E501	E501 - Vitamin A deficiency with Bitot's spot and conjunctival xerosis	Vitamin deficiency
E502	E502 - Vitamin A deficiency with corneal xerosis	Vitamin deficiency
E503	E503 - Vitamin A deficiency with corneal ulceration and xerosis	Vitamin deficiency
E504	E504 - Vitamin A deficiency with keratomalacia	Vitamin deficiency
E506	E506 - Vitamin A deficiency with xerophthalmic scars of cornea	Vitamin deficiency
E507	E507 - Other ocular manifestations of vitamin A deficiency	Vitamin deficiency
E508	E508 - Other manifestations of vitamin A deficiency	Vitamin deficiency
E511	E511 - Beriberi	Vitamin deficiency
E538	E538 - Deficiency of other specified B group vitamins	Vitamin deficiency
E54X	E54X - Ascorbic acid deficiency	Vitamin deficiency
E639	E639 - Nutritional deficiency, unspecified	Vitamin deficiency
A047	A047 - Enterocolitis due to Clostridium difficile	Exclusion
A099	A099 - Gastroenteritis and colitis of unspecified origin	Exclusion

ICD10 Code	Description	Category
A150	A150 - Tuberculosis of lung, confirmed by sputum microscopy with or without culture	Exclusion
A151	A151 - Tuberculosis of lung, confirmed by culture only	Exclusion
A152	A152 - Tuberculosis of lung, confirmed histologically	Exclusion
A153	A153 - Tuberculosis of lung, confirmed by unspecified means	Exclusion
A154	A154 - Tuberculosis of intrathoracic lymph nodes, confirmed bacteriologically and histologically	Exclusion
A155	A155 - Tuberculosis of larynx, trachea and bronchus, confirmed bacteriologically and histologically	Exclusion
A156	A156 - Tuberculous pleurisy, confirmed bacteriologically and histologically	Exclusion
A157	A157 - Primary respiratory tuberculosis, confirmed bacteriologically and histologically	Exclusion
A158	A158 - Other respiratory tuberculosis, confirmed bacteriologically and histologically	Exclusion
A159	A159 - Respiratory tuberculosis unspecified, confirmed bacteriologically and histologically	Exclusion
A160	A160 - Tuberculosis of lung, bacteriologically and histologically negative	Exclusion
A161	A161 - Tuberculosis of lung, bacteriological and histological examination not done	Exclusion
A162	A162 - Tuberculosis of lung, without mention of bacteriological or histological confirmation	Exclusion
A163	A163 - Tuberculosis of intrathoracic lymph nodes, without mention of bacteriological or histological confirmation	Exclusion
A164	A164 - Tuberculosis of larynx, trachea and bronchus, without mention of bacteriological or histological confirmation	Exclusion
A165	A165 - Tuberculous pleurisy, without mention of bacteriological or histological confirmation	Exclusion
A167	A167 - Primary respiratory tuberculosis without mention of bacteriological or histological confirmation	Exclusion
A168	A168 - Other respiratory tuberculosis, without mention of bacteriological or histological confirmation	Exclusion
A169	A169 - Respiratory tuberculosis unspecified, without mention of bacteriological or histological confirmation	Exclusion
A170	A170 - Tuberculous meningitis	Exclusion
A171	A171 - Meningeal tuberculoma	Exclusion
A178	A178 - Other tuberculosis of nervous system	Exclusion
A179	A179 - Tuberculosis of nervous system, unspecified	Exclusion
A180	A180 - Tuberculosis of bones and joints	Exclusion
A181	A181 - Tuberculosis of genitourinary system	Exclusion
A182	A182 - Tuberculous peripheral lymphadenopathy	Exclusion
A183	A183 - Tuberculosis of intestines, peritoneum and mesenteric glands	Exclusion
A184	A184 - Tuberculosis of skin and subcutaneous tissue	Exclusion
A185	A185 - Tuberculosis of eye	Exclusion

ICD10 Code	Description	Category
A186	A186 - Tuberculosis of ear	Exclusion
A187	A187 - Tuberculosis of adrenal glands	Exclusion
A188	A188 - Tuberculosis of other specified organs	Exclusion
A190	A190 - Acute miliary tuberculosis of a single specified site	Exclusion
A191	A191 - Acute miliary tuberculosis of multiple sites	Exclusion
A192	A192 - Acute miliary tuberculosis, unspecified	Exclusion
A198	A198 - Other miliary tuberculosis	Exclusion
A199	A199 - Miliary tuberculosis, unspecified	Exclusion
A415	A415 - Sepsis due to other Gram-negative organisms	Exclusion
A419	A419 - Sepsis, unspecified	Exclusion
B200	B200 - HIV disease resulting in mycobacterial infection	Exclusion
B201	B201 - HIV disease resulting in other bacterial infections	Exclusion
B202	B202 - HIV disease resulting in cytomegaloviral disease	Exclusion
B203	B203 - HIV disease resulting in other viral infections	Exclusion
B204	B204 - HIV disease resulting in candidiasis	Exclusion
B205	B205 - HIV disease resulting in other mycoses	Exclusion
B206	B206 - HIV disease resulting in Pneumocystis jirovecii pneumonia	Exclusion
B207	B207 - HIV disease resulting in multiple infections	Exclusion
B208	B208 - HIV disease resulting in other infectious and parasitic diseases	Exclusion
B209	B209 - HIV disease resulting in unspecified infectious or parasitic disease	Exclusion
B220	B220 - HIV disease resulting in encephalopathy	Exclusion
B238	B238 - HIV disease resulting in other specified conditions	Exclusion
B24X	B24X - Unspecified human immunodeficiency virus [HIV] disease	Exclusion
C000	C000 - Malignant neoplasm: External upper lip	Exclusion
C001	C001 - Malignant neoplasm: External lower lip	Exclusion
C002	C002 - Malignant neoplasm: External lip, unspecified	Exclusion
C003	C003 - Malignant neoplasm: Upper lip, inner aspect	Exclusion
C004	C004 - Malignant neoplasm: Lower lip, inner aspect	Exclusion
C005	C005 - Malignant neoplasm: Lip, unspecified, inner aspect	Exclusion

ICD10 Code	Description	Category
C006	C006 - Malignant neoplasm: Commissure of lip	Exclusion
C008	C008 - Malignant neoplasm: Overlapping lesion of lip	Exclusion
C009	C009 - Malignant neoplasm: Lip, unspecified	Exclusion
C01X	C01X - Malignant neoplasm of base of tongue	Exclusion
C020	C020 - Malignant neoplasm: Dorsal surface of tongue	Exclusion
C021	C021 - Malignant neoplasm: Border of tongue	Exclusion
C022	C022 - Malignant neoplasm: Ventral surface of tongue	Exclusion
C023	C023 - Malignant neoplasm: Anterior two-thirds of tongue, part unspecified	Exclusion
C024	C024 - Malignant neoplasm: Lingual tonsil	Exclusion
C028	C028 - Malignant neoplasm: Overlapping lesion of tongue	Exclusion
C029	C029 - Malignant neoplasm: Tongue, unspecified	Exclusion
C030	C030 - Malignant neoplasm: Upper gum	Exclusion
C031	C031 - Malignant neoplasm: Lower gum	Exclusion
C039	C039 - Malignant neoplasm: Gum, unspecified	Exclusion
C040	C040 - Malignant neoplasm: Anterior floor of mouth	Exclusion
C041	C041 - Malignant neoplasm: Lateral floor of mouth	Exclusion
C048	C048 - Malignant neoplasm: Overlapping lesion of floor of mouth	Exclusion
C049	C049 - Malignant neoplasm: Floor of mouth, unspecified	Exclusion
C050	C050 - Malignant neoplasm: Hard palate	Exclusion
C051	C051 - Malignant neoplasm: Soft palate	Exclusion
C052	C052 - Malignant neoplasm: Uvula	Exclusion
C058	C058 - Malignant neoplasm: Overlapping lesion of palate	Exclusion
C059	C059 - Malignant neoplasm: Palate, unspecified	Exclusion
C060	C060 - Malignant neoplasm: Cheek mucosa	Exclusion
C061	C061 - Malignant neoplasm: Vestibule of mouth	Exclusion
C062	C062 - Malignant neoplasm: Retromolar area	Exclusion
C068	C068 - Malignant neoplasm: Overlapping lesion of other and unspecified parts of mouth	Exclusion
C069	C069 - Malignant neoplasm: Mouth, unspecified	Exclusion
C07X	C07X - Malignant neoplasm of parotid gland	Exclusion

ICD10 Code	Description	Category
C080	C080 - Malignant neoplasm: Submandibular gland	Exclusion
C081	C081 - Malignant neoplasm: Sublingual gland	Exclusion
C088	C088 - Malignant neoplasm: Overlapping lesion of major salivary glands	Exclusion
C089	C089 - Malignant neoplasm: Major salivary gland, unspecified	Exclusion
C090	C090 - Malignant neoplasm: Tonsillar fossa	Exclusion
C091	C091 - Malignant neoplasm: Tonsillar pillar (anterior)(posterior)	Exclusion
C098	C098 - Malignant neoplasm: Overlapping lesion of tonsil	Exclusion
C099	C099 - Malignant neoplasm: Tonsil, unspecified	Exclusion
C100	C100 - Malignant neoplasm: Vallecula	Exclusion
C101	C101 - Malignant neoplasm: Anterior surface of epiglottis	Exclusion
C102	C102 - Malignant neoplasm: Lateral wall of oropharynx	Exclusion
C103	C103 - Malignant neoplasm: Posterior wall of oropharynx	Exclusion
C104	C104 - Malignant neoplasm: Branchial cleft	Exclusion
C108	C108 - Malignant neoplasm: Overlapping lesion of oropharynx	Exclusion
C109	C109 - Malignant neoplasm: Oropharynx, unspecified	Exclusion
C110	C110 - Malignant neoplasm: Superior wall of nasopharynx	Exclusion
C111	C111 - Malignant neoplasm: Posterior wall of nasopharynx	Exclusion
C112	C112 - Malignant neoplasm: Lateral wall of nasopharynx	Exclusion
C113	C113 - Malignant neoplasm: Anterior wall of nasopharynx	Exclusion
C118	C118 - Malignant neoplasm: Overlapping lesion of nasopharynx	Exclusion
C119	C119 - Malignant neoplasm: Nasopharynx, unspecified	Exclusion
C12X	C12X - Malignant neoplasm of piriform sinus	Exclusion
C130	C130 - Malignant neoplasm: Postcricoid region	Exclusion
C131	C131 - Malignant neoplasm: Aryepiglottic fold, hypopharyngeal aspect	Exclusion
C132	C132 - Malignant neoplasm: Posterior wall of hypopharynx	Exclusion
C138	C138 - Malignant neoplasm: Overlapping lesion of hypopharynx	Exclusion
C139	C139 - Malignant neoplasm: Hypopharynx, unspecified	Exclusion
C140	C140 - Malignant neoplasm: Pharynx, unspecified	Exclusion
C142	C142 - Malignant neoplasm: Waldeyer's ring	Exclusion

ICD10 Code	Description	Category
C148	C148 - Malignant neoplasm: Overlapping lesion of lip, oral cavity and pharynx	Exclusion
C150	C150 - Malignant neoplasm: Cervical part of oesophagus	Exclusion
C151	C151 - Malignant neoplasm: Thoracic part of oesophagus	Exclusion
C152	C152 - Malignant neoplasm: Abdominal part of oesophagus	Exclusion
C153	C153 - Malignant neoplasm: Upper third of oesophagus	Exclusion
C154	C154 - Malignant neoplasm: Middle third of oesophagus	Exclusion
C155	C155 - Malignant neoplasm: Lower third of oesophagus	Exclusion
C158	C158 - Malignant neoplasm: Overlapping lesion of oesophagus	Exclusion
C159	C159 - Malignant neoplasm: Oesophagus, unspecified	Exclusion
C160	C160 - Malignant neoplasm: Cardia	Exclusion
C161	C161 - Malignant neoplasm: Fundus of stomach	Exclusion
C162	C162 - Malignant neoplasm: Body of stomach	Exclusion
C163	C163 - Malignant neoplasm: Pyloric antrum	Exclusion
C164	C164 - Malignant neoplasm: Pylorus	Exclusion
C165	C165 - Malignant neoplasm: Lesser curvature of stomach, unspecified	Exclusion
C166	C166 - Malignant neoplasm: Greater curvature of stomach, unspecified	Exclusion
C168	C168 - Malignant neoplasm: Overlapping lesion of stomach	Exclusion
C169	C169 - Malignant neoplasm: Stomach, unspecified	Exclusion
C170	C170 - Malignant neoplasm: Duodenum	Exclusion
C171	C171 - Malignant neoplasm: Jejunum	Exclusion
C172	C172 - Malignant neoplasm: Ileum	Exclusion
C173	C173 - Malignant neoplasm: Meckel's diverticulum	Exclusion
C178	C178 - Malignant neoplasm: Overlapping lesion of small intestine	Exclusion
C179	C179 - Malignant neoplasm: Small intestine, unspecified	Exclusion
C180	C180 - Malignant neoplasm: Caecum	Exclusion
C181	C181 - Malignant neoplasm: Appendix	Exclusion
C182	C182 - Malignant neoplasm: Ascending colon	Exclusion
C183	C183 - Malignant neoplasm: Hepatic flexure	Exclusion
C184	C184 - Malignant neoplasm: Transverse colon	Exclusion

ICD10 Code	Description	Category
C185	C185 - Malignant neoplasm: Splenic flexure	Exclusion
C186	C186 - Malignant neoplasm: Descending colon	Exclusion
C187	C187 - Malignant neoplasm: Sigmoid colon	Exclusion
C188	C188 - Malignant neoplasm: Overlapping lesion of colon	Exclusion
C189	C189 - Malignant neoplasm: Colon, unspecified	Exclusion
C19X	C19X - Malignant neoplasm of rectosigmoid junction	Exclusion
C20X	C20X - Malignant neoplasm of rectum	Exclusion
C210	C210 - Malignant neoplasm: Anus, unspecified	Exclusion
C211	C211 - Malignant neoplasm: Anal canal	Exclusion
C212	C212 - Malignant neoplasm: Cloacogenic zone	Exclusion
C218	C218 - Malignant neoplasm: Overlapping lesion of rectum, anus and anal canal	Exclusion
C220	C220 - Malignant neoplasm: Liver cell carcinoma	Exclusion
C221	C221 - Malignant neoplasm: Intrahepatic bile duct carcinoma	Exclusion
C222	C222 - Malignant neoplasm: Hepatoblastoma	Exclusion
C223	C223 - Malignant neoplasm: Angiosarcoma of liver	Exclusion
C224	C224 - Malignant neoplasm: Other sarcomas of liver	Exclusion
C227	C227 - Malignant neoplasm: Other specified carcinomas of liver	Exclusion
C229	C229 - Malignant neoplasm: Liver, unspecified	Exclusion
C23X	C23X - Malignant neoplasm of gallbladder	Exclusion
C240	C240 - Malignant neoplasm: Extrahepatic bile duct	Exclusion
C241	C241 - Malignant neoplasm: Ampulla of Vater	Exclusion
C248	C248 - Malignant neoplasm: Overlapping lesion of biliary tract	Exclusion
C249	C249 - Malignant neoplasm: Biliary tract, unspecified	Exclusion
C250	C250 - Malignant neoplasm: Head of pancreas	Exclusion
C251	C251 - Malignant neoplasm: Body of pancreas	Exclusion
C252	C252 - Malignant neoplasm: Tail of pancreas	Exclusion
C253	C253 - Malignant neoplasm: Pancreatic duct	Exclusion
C254	C254 - Malignant neoplasm: Endocrine pancreas	Exclusion
C257	C257 - Malignant neoplasm: Other parts of pancreas	Exclusion

ICD10 Code	Description	Category
C258	C258 - Malignant neoplasm: Overlapping lesion of pancreas	Exclusion
C259	C259 - Malignant neoplasm: Pancreas, unspecified	Exclusion
C260	C260 - Malignant neoplasm: Intestinal tract, part unspecified	Exclusion
C261	C261 - Malignant neoplasm: Spleen	Exclusion
C268	C268 - Malignant neoplasm: Overlapping lesion of digestive system	Exclusion
C269	C269 - Malignant neoplasm: Ill-defined sites within the digestive system	Exclusion
C300	C300 - Malignant neoplasm: Nasal cavity	Exclusion
C301	C301 - Malignant neoplasm: Middle ear	Exclusion
C310	C310 - Malignant neoplasm: Maxillary sinus	Exclusion
C311	C311 - Malignant neoplasm: Ethmoidal sinus	Exclusion
C312	C312 - Malignant neoplasm: Frontal sinus	Exclusion
C313	C313 - Malignant neoplasm: Sphenoidal sinus	Exclusion
C318	C318 - Malignant neoplasm: Overlapping lesion of accessory sinuses	Exclusion
C319	C319 - Malignant neoplasm: Accessory sinus, unspecified	Exclusion
C320	C320 - Malignant neoplasm: Glottis	Exclusion
C321	C321 - Malignant neoplasm: Supraglottis	Exclusion
C322	C322 - Malignant neoplasm: Subglottis	Exclusion
C323	C323 - Malignant neoplasm: Laryngeal cartilage	Exclusion
C328	C328 - Malignant neoplasm: Overlapping lesion of larynx	Exclusion
C329	C329 - Malignant neoplasm: Larynx, unspecified	Exclusion
C33X	C33X - Malignant neoplasm of trachea	Exclusion
C340	C340 - Malignant neoplasm: Main bronchus	Exclusion
C341	C341 - Malignant neoplasm: Upper lobe, bronchus or lung	Exclusion
C342	C342 - Malignant neoplasm: Middle lobe, bronchus or lung	Exclusion
C343	C343 - Malignant neoplasm: Lower lobe, bronchus or lung	Exclusion
C348	C348 - Malignant neoplasm: Overlapping lesion of bronchus and lung	Exclusion
C349	C349 - Malignant neoplasm: Bronchus or lung, unspecified	Exclusion
C37X	C37X - Malignant neoplasm of thymus	Exclusion
C380	C380 - Malignant neoplasm: Heart	Exclusion

ICD10 Code	Description	Category
C381	C381 - Malignant neoplasm: Anterior mediastinum	Exclusion
C382	C382 - Malignant neoplasm: Posterior mediastinum	Exclusion
C383	C383 - Malignant neoplasm: Mediastinum, part unspecified	Exclusion
C384	C384 - Malignant neoplasm: Pleura	Exclusion
C388	C388 - Malignant neoplasm: Overlapping lesion of heart, mediastinum and pleura	Exclusion
C390	C390 - Malignant neoplasm: Upper respiratory tract, part unspecified	Exclusion
C398	C398 - Malignant neoplasm: Overlapping lesion of respiratory and intrathoracic organs	Exclusion
C399	C399 - Malignant neoplasm: Ill-defined sites within the respiratory system	Exclusion
C400	C400 - Malignant neoplasm: Scapula and long bones of upper limb	Exclusion
C401	C401 - Malignant neoplasm: Short bones of upper limb	Exclusion
C402	C402 - Malignant neoplasm: Long bones of lower limb	Exclusion
C403	C403 - Malignant neoplasm: Short bones of lower limb	Exclusion
C408	C408 - Malignant neoplasm: Overlapping lesion of bone and articular cartilage of limbs	Exclusion
C409	C409 - Malignant neoplasm: Bone and articular cartilage of limb, unspecified	Exclusion
C410	C410 - Malignant neoplasm: Bones of skull and face	Exclusion
C411	C411 - Malignant neoplasm: Mandible	Exclusion
C412	C412 - Malignant neoplasm: Vertebral column	Exclusion
C413	C413 - Malignant neoplasm: Ribs, sternum and clavicle	Exclusion
C414	C414 - Malignant neoplasm: Pelvic bones, sacrum and coccyx	Exclusion
C418	C418 - Malignant neoplasm: Overlapping lesion of bone and articular cartilage	Exclusion
C419	C419 - Malignant neoplasm: Bone and articular cartilage, unspecified	Exclusion
C430	C430 - Malignant neoplasm: Malignant melanoma of lip	Exclusion
C431	C431 - Malignant neoplasm: Malignant melanoma of eyelid, including canthus	Exclusion
C432	C432 - Malignant neoplasm: Malignant melanoma of ear and external auricular canal	Exclusion
C433	C433 - Malignant neoplasm: Malignant melanoma of other and unspecified parts of face	Exclusion
C434	C434 - Malignant neoplasm: Malignant melanoma of scalp and neck	Exclusion
C435	C435 - Malignant neoplasm: Malignant melanoma of trunk	Exclusion
C436	C436 - Malignant neoplasm: Malignant melanoma of upper limb, including shoulder	Exclusion
C437	C437 - Malignant neoplasm: Malignant melanoma of lower limb, including hip	Exclusion

ICD10 Code	Description	Category
C438	C438 - Malignant neoplasm: Overlapping malignant melanoma of skin	Exclusion
C439	C439 - Malignant neoplasm: Malignant melanoma of skin, unspecified	Exclusion
C440	C440 - Malignant neoplasm: Skin of lip	Exclusion
C441	C441 - Malignant neoplasm: Skin of eyelid, including canthus	Exclusion
C442	C442 - Malignant neoplasm: Skin of ear and external auricular canal	Exclusion
C443	C443 - Malignant neoplasm: Skin of other and unspecified parts of face	Exclusion
C444	C444 - Malignant neoplasm: Skin of scalp and neck	Exclusion
C445	C445 - Malignant neoplasm: Skin of trunk	Exclusion
C446	C446 - Malignant neoplasm: Skin of upper limb, including shoulder	Exclusion
C447	C447 - Malignant neoplasm: Skin of lower limb, including hip	Exclusion
C448	C448 - Malignant neoplasm: Overlapping lesion of skin	Exclusion
C449	C449 - Malignant neoplasm: Malignant neoplasm of skin, unspecified	Exclusion
C450	C450 - Mesothelioma of pleura	Exclusion
C451	C451 - Mesothelioma of peritoneum	Exclusion
C452	C452 - Mesothelioma of pericardium	Exclusion
C457	C457 - Mesothelioma of other sites	Exclusion
C459	C459 - Mesothelioma, unspecified	Exclusion
C460	C460 - Kaposi sarcoma of skin	Exclusion
C461	C461 - Kaposi sarcoma of soft tissue	Exclusion
C462	C462 - Kaposi sarcoma of palate	Exclusion
C463	C463 - Kaposi sarcoma of lymph nodes	Exclusion
C467	C467 - Kaposi sarcoma of other sites	Exclusion
C468	C468 - Kaposi sarcoma of multiple organs	Exclusion
C469	C469 - Kaposi sarcoma, unspecified	Exclusion
C470	C470 - Malignant neoplasm: Peripheral nerves of head, face and neck	Exclusion
C471	C471 - Malignant neoplasm: Peripheral nerves of upper limb, including shoulder	Exclusion
C472	C472 - Malignant neoplasm: Peripheral nerves of lower limb, including hip	Exclusion
C473	C473 - Malignant neoplasm: Peripheral nerves of thorax	Exclusion
C474	C474 - Malignant neoplasm: Peripheral nerves of abdomen	Exclusion

ICD10 Code	Description	Category
C475	C475 - Malignant neoplasm: Peripheral nerves of pelvis	Exclusion
C476	C476 - Malignant neoplasm: Peripheral nerves of trunk, unspecified	Exclusion
C478	C478 - Malignant neoplasm: Overlapping lesion of peripheral nerves and autonomic nervous system	Exclusion
C479	C479 - Malignant neoplasm: Peripheral nerves and autonomic nervous system, unspecified	Exclusion
C480	C480 - Malignant neoplasm: Retroperitoneum	Exclusion
C481	C481 - Malignant neoplasm: Specified parts of peritoneum	Exclusion
C482	C482 - Malignant neoplasm: Peritoneum, unspecified	Exclusion
C488	C488 - Malignant neoplasm: Overlapping lesion of retroperitoneum and peritoneum	Exclusion
C490	C490 - Malignant neoplasm: Connective and soft tissue of head, face and neck	Exclusion
C491	C491 - Malignant neoplasm: Connective and soft tissue of upper limb, including shoulder	Exclusion
C492	C492 - Malignant neoplasm: Connective and soft tissue of lower limb, including hip	Exclusion
C493	C493 - Malignant neoplasm: Connective and soft tissue of thorax	Exclusion
C494	C494 - Malignant neoplasm: Connective and soft tissue of abdomen	Exclusion
C495	C495 - Malignant neoplasm: Connective and soft tissue of pelvis	Exclusion
C496	C496 - Malignant neoplasm: Connective and soft tissue of trunk, unspecified	Exclusion
C498	C498 - Malignant neoplasm: Overlapping lesion of connective and soft tissue	Exclusion
C499	C499 - Malignant neoplasm: Connective and soft tissue, unspecified	Exclusion
C500	C500 - Malignant neoplasm: Nipple and areola	Exclusion
C501	C501 - Malignant neoplasm: Central portion of breast	Exclusion
C502	C502 - Malignant neoplasm: Upper-inner quadrant of breast	Exclusion
C503	C503 - Malignant neoplasm: Lower-inner quadrant of breast	Exclusion
C504	C504 - Malignant neoplasm: Upper-outer quadrant of breast	Exclusion
C505	C505 - Malignant neoplasm: Lower-outer quadrant of breast	Exclusion
C506	C506 - Malignant neoplasm: Axillary tail of breast	Exclusion
C508	C508 - Malignant neoplasm: Overlapping lesion of breast	Exclusion
C509	C509 - Malignant neoplasm: Breast, unspecified	Exclusion
C510	C510 - Malignant neoplasm: Labium majus	Exclusion
C511	C511 - Malignant neoplasm: Labium minus	Exclusion
C512	C512 - Malignant neoplasm: Clitoris	Exclusion

ICD10 Code	Description	Category
C518	C518 - Malignant neoplasm: Overlapping lesion of vulva	Exclusion
C519	C519 - Malignant neoplasm: Vulva, unspecified	Exclusion
C52X	C52X - Malignant neoplasm of vagina	Exclusion
C530	C530 - Malignant neoplasm: Endocervix	Exclusion
C531	C531 - Malignant neoplasm: Exocervix	Exclusion
C538	C538 - Malignant neoplasm: Overlapping lesion of cervix uteri	Exclusion
C539	C539 - Malignant neoplasm: Cervix uteri, unspecified	Exclusion
C540	C540 - Malignant neoplasm: Isthmus uteri	Exclusion
C541	C541 - Malignant neoplasm: Endometrium	Exclusion
C542	C542 - Malignant neoplasm: Myometrium	Exclusion
C543	C543 - Malignant neoplasm: Fundus uteri	Exclusion
C548	C548 - Malignant neoplasm: Overlapping lesion of corpus uteri	Exclusion
C549	C549 - Malignant neoplasm: Corpus uteri, unspecified	Exclusion
C55X	C55X - Malignant neoplasm of uterus, part unspecified	Exclusion
C56X	C56X - Malignant neoplasm of ovary	Exclusion
C570	C570 - Malignant neoplasm: Fallopian tube	Exclusion
C571	C571 - Malignant neoplasm: Broad ligament	Exclusion
C572	C572 - Malignant neoplasm: Round ligament	Exclusion
C573	C573 - Malignant neoplasm: Parametrium	Exclusion
C574	C574 - Malignant neoplasm: Uterine adnexa, unspecified	Exclusion
C577	C577 - Malignant neoplasm: Other specified female genital organs	Exclusion
C578	C578 - Malignant neoplasm: Overlapping lesion of female genital organs	Exclusion
C579	C579 - Malignant neoplasm: Female genital organ, unspecified	Exclusion
C58X	C58X - Malignant neoplasm of placenta	Exclusion
C600	C600 - Malignant neoplasm: Prepuce	Exclusion
C601	C601 - Malignant neoplasm: Glans penis	Exclusion
C602	C602 - Malignant neoplasm: Body of penis	Exclusion
C608	C608 - Malignant neoplasm: Overlapping lesion of penis	Exclusion
C609	C609 - Malignant neoplasm: Penis, unspecified	Exclusion

ICD10 Code	Description	Category
C61X	C61X - Malignant neoplasm of prostate	Exclusion
C620	C620 - Malignant neoplasm: Undescended testis	Exclusion
C621	C621 - Malignant neoplasm: Descended testis	Exclusion
C629	C629 - Malignant neoplasm: Testis, unspecified	Exclusion
C630	C630 - Malignant neoplasm: Epididymis	Exclusion
C631	C631 - Malignant neoplasm: Spermatic cord	Exclusion
C632	C632 - Malignant neoplasm: Scrotum	Exclusion
C637	C637 - Malignant neoplasm: Other specified male genital organs	Exclusion
C638	C638 - Malignant neoplasm: Overlapping lesion of male genital organs	Exclusion
C639	C639 - Malignant neoplasm: Male genital organ, unspecified	Exclusion
C64X	C64X - Malignant neoplasm of kidney, except renal pelvis	Exclusion
C65X	C65X - Malignant neoplasm of renal pelvis	Exclusion
C66X	C66X - Malignant neoplasm of ureter	Exclusion
C670	C670 - Malignant neoplasm: Trigone of bladder	Exclusion
C671	C671 - Malignant neoplasm: Dome of bladder	Exclusion
C672	C672 - Malignant neoplasm: Lateral wall of bladder	Exclusion
C673	C673 - Malignant neoplasm: Anterior wall of bladder	Exclusion
C674	C674 - Malignant neoplasm: Posterior wall of bladder	Exclusion
C675	C675 - Malignant neoplasm: Bladder neck	Exclusion
C676	C676 - Malignant neoplasm: Ureteric orifice	Exclusion
C677	C677 - Malignant neoplasm: Urachus	Exclusion
C678	C678 - Malignant neoplasm: Overlapping lesion of bladder	Exclusion
C679	C679 - Malignant neoplasm: Bladder, unspecified	Exclusion
C680	C680 - Malignant neoplasm: Urethra	Exclusion
C681	C681 - Malignant neoplasm: Paraurethral gland	Exclusion
C688	C688 - Malignant neoplasm: Overlapping lesion of urinary organs	Exclusion
C689	C689 - Malignant neoplasm: Urinary organ, unspecified	Exclusion
C690	C690 - Malignant neoplasm: Conjunctiva	Exclusion
C691	C691 - Malignant neoplasm: Cornea	Exclusion

ICD10 Code	Description	Category
C692	C692 - Malignant neoplasm: Retina	Exclusion
C693	C693 - Malignant neoplasm: Choroid	Exclusion
C694	C694 - Malignant neoplasm: Ciliary body	Exclusion
C695	C695 - Malignant neoplasm: Lacrimal gland and duct	Exclusion
C696	C696 - Malignant neoplasm: Orbit	Exclusion
C698	C698 - Malignant neoplasm: Overlapping lesion of eye and adnexa	Exclusion
C699	C699 - Malignant neoplasm: Eye, unspecified	Exclusion
C700	C700 - Malignant neoplasm: Cerebral meninges	Exclusion
C701	C701 - Malignant neoplasm: Spinal meninges	Exclusion
C709	C709 - Malignant neoplasm: Meninges, unspecified	Exclusion
C710	C710 - Malignant neoplasm: Cerebrum, except lobes and ventricles	Exclusion
C711	C711 - Malignant neoplasm: Frontal lobe	Exclusion
C712	C712 - Malignant neoplasm: Temporal lobe	Exclusion
C713	C713 - Malignant neoplasm: Parietal lobe	Exclusion
C714	C714 - Malignant neoplasm: Occipital lobe	Exclusion
C715	C715 - Malignant neoplasm: Cerebral ventricle	Exclusion
C716	C716 - Malignant neoplasm: Cerebellum	Exclusion
C717	C717 - Malignant neoplasm: Brain stem	Exclusion
C718	C718 - Malignant neoplasm: Overlapping lesion of brain	Exclusion
C719	C719 - Malignant neoplasm: Brain, unspecified	Exclusion
C720	C720 - Malignant neoplasm: Spinal cord	Exclusion
C721	C721 - Malignant neoplasm: Cauda equina	Exclusion
C722	C722 - Malignant neoplasm: Olfactory nerve	Exclusion
C723	C723 - Malignant neoplasm: Optic nerve	Exclusion
C724	C724 - Malignant neoplasm: Acoustic nerve	Exclusion
C725	C725 - Malignant neoplasm: Other and unspecified cranial nerves	Exclusion
C728	C728 - Malignant neoplasm: Overlapping lesion of brain and other parts of central nervous system	Exclusion
C729	C729 - Malignant neoplasm: Central nervous system, unspecified	Exclusion
C73X	C73X - Malignant neoplasm of thyroid gland	Exclusion

ICD10 Code	Description	Category
C740	C740 - Malignant neoplasm: Cortex of adrenal gland	Exclusion
C741	C741 - Malignant neoplasm: Medulla of adrenal gland	Exclusion
C749	C749 - Malignant neoplasm: Adrenal gland, unspecified	Exclusion
C750	C750 - Malignant neoplasm: Parathyroid gland	Exclusion
C751	C751 - Malignant neoplasm: Pituitary gland	Exclusion
C752	C752 - Malignant neoplasm: Craniopharyngeal duct	Exclusion
C753	C753 - Malignant neoplasm: Pineal gland	Exclusion
C754	C754 - Malignant neoplasm: Carotid body	Exclusion
C755	C755 - Malignant neoplasm: Aortic body and other paraganglia	Exclusion
C758	C758 - Malignant neoplasm: Pluriglandular involvement, unspecified	Exclusion
C759	C759 - Malignant neoplasm: Endocrine gland, unspecified	Exclusion
C760	C760 - Malignant neoplasm of other and ill-defined sites: Head, face and neck	Exclusion
C761	C761 - Malignant neoplasm of other and ill-defined sites: Thorax	Exclusion
C762	C762 - Malignant neoplasm of other and ill-defined sites: Abdomen	Exclusion
C763	C763 - Malignant neoplasm of other and ill-defined sites: Pelvis	Exclusion
C764	C764 - Malignant neoplasm of other and ill-defined sites: Upper limb	Exclusion
C765	C765 - Malignant neoplasm of other and ill-defined sites: Lower limb	Exclusion
C767	C767 - Malignant neoplasm of other and ill-defined sites: Other ill-defined sites	Exclusion
C768	C768 - Malignant neoplasm of other and ill-defined sites: Overlapping lesion of other and ill-defined sites	Exclusion
C770	C770 - Secondary and unspecified malignant neoplasm: Lymph nodes of head, face and neck	Exclusion
C771	C771 - Secondary and unspecified malignant neoplasm: Intrathoracic lymph nodes	Exclusion
C772	C772 - Secondary and unspecified malignant neoplasm: Intra-abdominal lymph nodes	Exclusion
C773	C773 - Secondary and unspecified malignant neoplasm: Axillary and upper limb lymph nodes	Exclusion
C774	C774 - Secondary and unspecified malignant neoplasm: Inguinal and lower limb lymph nodes	Exclusion
C775	C775 - Secondary and unspecified malignant neoplasm: Intrapelvic lymph nodes	Exclusion
C778	C778 - Secondary and unspecified malignant neoplasm: Lymph nodes of multiple regions	Exclusion
C779	C779 - Secondary and unspecified malignant neoplasm: Lymph node, unspecified	Exclusion
C780	C780 - Secondary malignant neoplasm of lung	Exclusion
C781	C781 - Secondary malignant neoplasm of mediastinum	Exclusion

ICD10 Code	Description	Category
C782	C782 - Secondary malignant neoplasm of pleura	Exclusion
C783	C783 - Secondary malignant neoplasm of other and unspecified respiratory organs	Exclusion
C784	C784 - Secondary malignant neoplasm of small intestine	Exclusion
C785	C785 - Secondary malignant neoplasm of large intestine and rectum	Exclusion
C786	C786 - Secondary malignant neoplasm of retroperitoneum and peritoneum	Exclusion
C787	C787 - Secondary malignant neoplasm of liver and intrahepatic bile duct	Exclusion
C788	C788 - Secondary malignant neoplasm of other and unspecified digestive organs	Exclusion
C790	C790 - Secondary malignant neoplasm of kidney and renal pelvis	Exclusion
C791	C791 - Secondary malignant neoplasm of bladder and other and unspecified urinary organs	Exclusion
C792	C792 - Secondary malignant neoplasm of skin	Exclusion
C793	C793 - Secondary malignant neoplasm of brain and cerebral meninges	Exclusion
C794	C794 - Secondary malignant neoplasm of other and unspecified parts of nervous system	Exclusion
C795	C795 - Secondary malignant neoplasm of bone and bone marrow	Exclusion
C796	C796 - Secondary malignant neoplasm of ovary	Exclusion
C797	C797 - Secondary malignant neoplasm of adrenal gland	Exclusion
C798	C798 - Secondary malignant neoplasm of other specified sites	Exclusion
C799	C799 - Secondary malignant neoplasm, unspecified site	Exclusion
C800	C800 - Malignant neoplasm, primary site unknown, so stated	Exclusion
C809	C809 - Malignant neoplasm, unspecified	Exclusion
C80X	C80X - Malignant neoplasm without specification of site	Exclusion
C810	C810 - Nodular lymphocyte predominant Hodgkin lymphoma	Exclusion
C811	C811 - Nodular sclerosis classical Hodgkin lymphoma	Exclusion
C812	C812 - Mixed cellularity classical Hodgkin lymphoma	Exclusion
C813	C813 - Lymphocyte depleted classical Hodgkin lymphoma	Exclusion
C814	C814 - Lymphocyte-rich classical Hodgkin lymphoma	Exclusion
C817	C817 - Other classical Hodgkin lymphoma	Exclusion
C819	C819 - Hodgkin lymphoma, unspecified	Exclusion
C820	C820 - Follicular lymphoma grade I	Exclusion
C821	C821 - Follicular lymphoma grade II	Exclusion

ICD10 Code	Description	Category
C822	C822 - Follicular lymphoma grade III, unspecified	Exclusion
C823	C823 - Follicular lymphoma grade IIIa	Exclusion
C824	C824 - Follicular lymphoma grade IIIb	Exclusion
C825	C825 - Diffuse follicle centre lymphoma	Exclusion
C826	C826 - Cutaneous follicle centre lymphoma	Exclusion
C827	C827 - Other types of follicular lymphoma	Exclusion
C829	C829 - Follicular lymphoma, unspecified	Exclusion
C830	C830 - Small cell B-cell lymphoma	Exclusion
C831	C831 - Mantle cell lymphoma	Exclusion
C832	C832 - Mixed small and large cell (diffuse) non-Hodgkins lymphoma	Exclusion
C833	C833 - Diffuse large B-cell lymphoma	Exclusion
C834	C834 - Immunoblastic (diffuse) non-Hodgkins lymphoma	Exclusion
C835	C835 - Lymphoblastic (diffuse) lymphoma	Exclusion
C836	C836 - Undifferentiated (diffuse) non-Hodgkins lymphoma	Exclusion
C837	C837 - Burkitt lymphoma	Exclusion
C838	C838 - Other non-follicular lymphoma	Exclusion
C839	C839 - Non-follicular (diffuse) lymphoma, unspecified	Exclusion
C840	C840 - Mycosis fungoides	Exclusion
C841	C841 - Sézary disease	Exclusion
C842	C842 - T-zone lymphoma	Exclusion
C843	C843 - Lymphoepithelioid lymphoma	Exclusion
C844	C844 - Peripheral T-cell lymphoma, not elsewhere classified	Exclusion
C845	C845 - Other mature T/NK-cell lymphomas	Exclusion
C846	C846 - Anaplastic large cell lymphoma, ALK-positive	Exclusion
C847	C847 - Anaplastic large cell lymphoma, ALK-negative	Exclusion
C848	C848 - Cutaneous T-cell lymphoma, unspecified	Exclusion
C849	C849 - Mature T/NK-cell lymphoma, unspecified	Exclusion
C850	C850 - Lymphosarcoma	Exclusion
C851	C851 - B-cell lymphoma, unspecified	Exclusion

ICD10 Code	Description	Category
C852	C852 - Mediastinal (thymic) large B-cell lymphoma	Exclusion
C857	C857 - Other specified types of non-Hodgkin lymphoma	Exclusion
C859	C859 - Non-Hodgkin lymphoma, unspecified	Exclusion
C860	C860 - Extranodal NK/T-cell lymphoma, nasal type	Exclusion
C861	C861 - Hepatosplenic T-cell lymphoma	Exclusion
C862	C862 - Enteropathy-type (intestinal) T-cell lymphoma	Exclusion
C863	C863 - Subcutaneous panniculitis-like T-cell lymphoma	Exclusion
C864	C864 - Blastic NK-cell lymphoma	Exclusion
C865	C865 - Angioimmunoblastic T-cell lymphoma	Exclusion
C866	C866 - Primary cutaneous CD30-positive T-cell proliferations	Exclusion
C880	C880 - Waldenström macroglobulinaemia	Exclusion
C881	C881 - Alpha heavy chain disease	Exclusion
C882	C882 - Other heavy chain disease	Exclusion
C883	C883 - Immunoproliferative small intestinal disease	Exclusion
C884	C884 - Extranodal marginal zone B-cell lymphoma of mucosa-associated lymphoid tissue [MALT-lymphoma]	Exclusion
C887	C887 - Other malignant immunoproliferative diseases	Exclusion
C889	C889 - Malignant immunoproliferative disease, unspecified	Exclusion
C900	C900 - Multiple myeloma	Exclusion
C901	C901 - Plasma cell leukaemia	Exclusion
C902	C902 - Extramedullary plasmacytoma	Exclusion
C903	C903 - Solitary plasmacytoma	Exclusion
C910	C910 - Acute lymphoblastic leukaemia [ALL]	Exclusion
C911	C911 - Chronic lymphocytic leukaemia of B-cell type	Exclusion
C912	C912 - Subacute lymphocytic leukaemia	Exclusion
C913	C913 - Prolymphocytic leukaemia of B-cell type	Exclusion
C914	C914 - Hairy-cell leukaemia	Exclusion
C915	C915 - Adult T-cell lymphoma/leukaemia (HTLV-1-associated)	Exclusion
C916	C916 - Prolymphocytic leukaemia of T-cell type	Exclusion
C917	C917 - Other lymphoid leukaemia	Exclusion

ICD10 Code	Description	Category
C918	C918 - Mature B-cell leukaemia Burkitt-type	Exclusion
C919	C919 - Lymphoid leukaemia, unspecified	Exclusion
C920	C920 - Acute myeloblastic leukaemia [AML]	Exclusion
C921	C921 - Chronic myeloid leukaemia [CML], BCR/ABL-positive	Exclusion
C922	C922 - Atypical chronic myeloid leukaemia, BCR/ABL-negative	Exclusion
C923	C923 - Myeloid sarcoma	Exclusion
C924	C924 - Acute promyelocytic leukaemia [PML]	Exclusion
C925	C925 - Acute myelomonocytic leukaemia	Exclusion
C926	C926 - Acute myeloid leukaemia with 11q23-abnormality	Exclusion
C927	C927 - Other myeloid leukaemia	Exclusion
C928	C928 - Acute myeloid leukaemia with multilineage dysplasia	Exclusion
C929	C929 - Myeloid leukaemia, unspecified	Exclusion
C930	C930 - Acute monoblastic/monocytic leukaemia	Exclusion
C931	C931 - Chronic myelomonocytic leukaemia	Exclusion
C932	C932 - Subacute monocytic leukaemia	Exclusion
C933	C933 - Juvenile myelomonocytic leukaemia	Exclusion
C937	C937 - Other monocytic leukaemia	Exclusion
C939	C939 - Monocytic leukaemia, unspecified	Exclusion
C940	C940 - Acute erythroid leukaemia	Exclusion
C941	C941 - Chronic erythraemia	Exclusion
C942	C942 - Acute megakaryoblastic leukaemia	Exclusion
C943	C943 - Mast cell leukaemia	Exclusion
C944	C944 - Acute panmyelosis with myelofibrosis	Exclusion
C945	C945 - Acute myelofibrosis	Exclusion
C946	C946 - Myelodysplastic and myeloproliferative disease, not elsewhere classified	Exclusion
C947	C947 - Other specified leukaemias	Exclusion
C950	C950 - Acute leukaemia of unspecified cell type	Exclusion
C951	C951 - Chronic leukaemia of unspecified cell type	Exclusion
C952	C952 - Subacute leukaemia of unspecified cell type	Exclusion

ICD10 Code	Description	Category
C957	C957 - Other leukaemia of unspecified cell type	Exclusion
C959	C959 - Leukaemia, unspecified	Exclusion
C960	C960 - Multifocal and multisystemic (disseminated) Langerhans-cell histiocytosis [Letterer-Siwe disease]	Exclusion
C961	C961 - Malignant histiocytosis	Exclusion
C962	C962 - Malignant mast cell tumour	Exclusion
C963	C963 - True histiocytic lymphoma	Exclusion
C964	C964 - Sarcoma of dendritic cells (accessory cells)	Exclusion
C965	C965 - Multifocal and unisystemic Langerhans-cell histiocytosis	Exclusion
C966	C966 - Unifocal Langerhans-cell histiocytosis	Exclusion
C967	C967 - Other specified malignant neoplasms of lymphoid, haematopoietic and related tissue	Exclusion
C968	C968 - Histiocytic sarcoma	Exclusion
C969	C969 - Malignant neoplasm of lymphoid, haematopoietic and related tissue, unspecified	Exclusion
C97X	C97X - Malignant neoplasms of independent (primary) multiple sites	Exclusion
D471	D471 - Chronic myeloproliferative disease	Exclusion
D70X	D70X - Agranulocytosis	Exclusion
E050	E050 - Thyrotoxicosis with diffuse goitre	Exclusion
E101	E101 - Insulin-dependent diabetes mellitus. With ketoacidosis	Exclusion
E162	E162 - Hypoglycaemia, unspecified	Exclusion
E840	E840 - Cystic fibrosis with pulmonary manifestations	Exclusion
E848	E848 - Cystic fibrosis with other manifestations	Exclusion
E849	E849 - Cystic fibrosis, unspecified	Exclusion
F019	F019 - Vascular dementia, unspecified	Exclusion
F03X	F03X - Unspecified dementia	Exclusion
F051	F051 - Delirium superimposed on dementia	Exclusion
F059	F059 - Delirium, unspecified	Exclusion
F200	F200 - Paranoid schizophrenia	Exclusion
F202	F202 - Catatonic schizophrenia	Exclusion
F209	F209 - Schizophrenia, unspecified	Exclusion
F220	F220 - Delusional disorder	Exclusion

ICD10 Code	Description	Category
F323	F323 - Severe depressive episode with psychotic symptoms	Exclusion
F500	F500 - Anorexia nervosa	Exclusion
F501	F501 - Atypical anorexia nervosa	Exclusion
F502	F502 - Bulimia nervosa	Exclusion
F503	F503 - Atypical bulimia nervosa	Exclusion
F504	F504 - Overeating associated with other psychological disturbances	Exclusion
F505	F505 - Vomiting associated with other psychological disturbances	Exclusion
F508	F508 - Other eating disorders	Exclusion
F509	F509 - Eating disorder, unspecified	Exclusion
G10X	G10X - Huntington's disease	Exclusion
G122	G122 - Motor neuron disease	Exclusion
G20X	G20X - Parkinson's disease	Exclusion
G309	G309 - Alzheimer's disease, unspecified	Exclusion
G319	G319 - Degenerative disease of nervous system, unspecified	Exclusion
G35X	G35X - Multiple sclerosis	Exclusion
H062	H062 - Dysthyroid exophthalmos	Exclusion
H251	H251 - Senile nuclear cataract	Exclusion
I219	I219 - Acute myocardial infarction, unspecified	Exclusion
I269	I269 - Pulmonary embolism without mention of acute cor pulmonale	Exclusion
I480	I480 - Paroxysmal atrial fibrillation	Exclusion
I481	I481 - Persistent atrial fibrillation	Exclusion
I482	I482 - Chronic atrial fibrillation	Exclusion
I483	I483 - Typical atrial flutter	Exclusion
I484	I484 - Atypical atrial flutter	Exclusion
I489	I489 - Atrial fibrillation and atrial flutter, unspecified	Exclusion
I48X	I48X - Atrial fibrillation and flutter	Exclusion
I500	I500 - Congestive heart failure	Exclusion
I501	I501 - Left ventricular failure	Exclusion
I64X	I64X - Stroke, not specified as haemorrhage or infarction	Exclusion

ICD10 Code	Description	Category
I739	I739 - Peripheral vascular disease, unspecified	Exclusion
I802	I802 - Phlebitis and thrombophlebitis of other deep vessels of lower extremities	Exclusion
I850	I850 - Oesophageal varices with bleeding	Exclusion
I951	I951 - Orthostatic hypotension	Exclusion
J180	J180 - Bronchopneumonia, unspecified	Exclusion
J189	J189 - Pneumonia, unspecified	Exclusion
J439	J439 - Emphysema, unspecified	Exclusion
J440	J440 - Chronic obstructive pulmonary disease with acute lower respiratory infection	Exclusion
J47X	J47X - Bronchiectasis	Exclusion
J690	J690 - Pneumonitis due to food and vomit	Exclusion
J869	J869 - Pyothorax without fistula	Exclusion
J90X	J90X - Pleural effusion, not elsewhere classified	Exclusion
J969	J969 - Respiratory failure, unspecified	Exclusion
K20X	K20X - Oesophagitis	Exclusion
K210	K210 - Gastro-oesophageal reflux disease with oesophagitis	Exclusion
K220	K220 - Achalasia of cardia	Exclusion
K221	K221 - Ulcer of oesophagus	Exclusion
K222	K222 - Oesophageal obstruction	Exclusion
K224	K224 - Dyskinesia of oesophagus	Exclusion
K228	K228 - Other specified diseases of oesophagus	Exclusion
K297	K297 - Gastritis, unspecified	Exclusion
K299	K299 - Gastroduodenitis, unspecified	Exclusion
K311	K311 - Adult hypertrophic pyloric stenosis	Exclusion
K315	K315 - Obstruction of duodenum	Exclusion
K318	K318 - Other specified diseases of stomach and duodenum	Exclusion
K500	K500 - Crohn's disease of small intestine	Exclusion
K501	K501 - Crohn's disease of large intestine	Exclusion
K508	K508 - Other Crohn's disease	Exclusion
K509	K509 - Crohn's disease, unspecified	Exclusion

ICD10 Code	Description	Category
K510	K510 - Ulcerative (chronic) pancolitis	Exclusion
K513	K513 - Ulcerative (chronic) rectosigmoiditis	Exclusion
K519	K519 - Ulcerative colitis, unspecified	Exclusion
K529	K529 - Noninfective gastroenteritis and colitis, unspecified	Exclusion
K550	K550 - Acute vascular disorders of intestine	Exclusion
K551	K551 - Chronic vascular disorders of intestine	Exclusion
K559	K559 - Vascular disorder of intestine, unspecified	Exclusion
K560	K560 - Paralytic ileus	Exclusion
K564	K564 - Other impaction of intestine	Exclusion
K565	K565 - Intestinal adhesions [bands] with obstruction	Exclusion
K566	K566 - Other and unspecified intestinal obstruction	Exclusion
K567	K567 - Ileus, unspecified	Exclusion
K573	K573 - Diverticular disease of large intestine without perforation or abscess	Exclusion
K598	K598 - Other specified functional intestinal disorders	Exclusion
K599	K599 - Functional intestinal disorder, unspecified	Exclusion
K631	K631 - Perforation of intestine (nontraumatic)	Exclusion
K632	K632 - Fistula of intestine	Exclusion
K638	K638 - Other specified diseases of intestine	Exclusion
K650	K650 - Acute peritonitis	Exclusion
K701	K701 - Alcoholic hepatitis	Exclusion
K703	K703 - Alcoholic cirrhosis of liver	Exclusion
K704	K704 - Alcoholic hepatic failure	Exclusion
K709	K709 - Alcoholic liver disease, unspecified	Exclusion
K729	K729 - Hepatic failure, unspecified	Exclusion
K743	K743 - Primary biliary cirrhosis	Exclusion
K831	K831 - Obstruction of bile duct	Exclusion
K859	K859 - Acute pancreatitis, unspecified	Exclusion
K861	K861 - Other chronic pancreatitis	Exclusion
K868	K868 - Other specified diseases of pancreas	Exclusion

ICD10 Code	Description	Category
K900	K900 - Coeliac disease	Exclusion
K904	K904 - Malabsorption due to intolerance, not elsewhere classified	Exclusion
K908	K908 - Other intestinal malabsorption	Exclusion
K909	K909 - Intestinal malabsorption, unspecified	Exclusion
K911	K911 - Postgastric surgery syndromes	Exclusion
K912	K912 - Postsurgical malabsorption, not elsewhere classified	Exclusion
K913	K913 - Postoperative intestinal obstruction	Exclusion
K914	K914 - Colostomy and enterostomy malfunction	Exclusion
K918	K918 - Other postprocedural disorders of digestive system, not elsewhere classified	Exclusion
K920	K920 - Haematemesis	Exclusion
K921	K921 - Melaena	Exclusion
K922	K922 - Gastrointestinal haemorrhage, unspecified	Exclusion
N179	N179 - Acute renal failure, unspecified	Exclusion
N180	N180 - End-stage renal disease	Exclusion
N185	N185 - Chronic kidney disease, stage 5	Exclusion
N390	N390 - Urinary tract infection, site not specified	Exclusion
O211	O211 - Hyperemesis gravidarum with metabolic disturbance	Exclusion
O992	O992 - Endocrine, nutritional and metabolic diseases complicating pregnancy, childbirth and the puerperium	Exclusion
R13X	R13X - Dysphagia	Exclusion
R18X	R18X - Ascites	Exclusion
R418	R418 - Other and unspecified symptoms and signs involving cognitive functions and awareness	Exclusion
R54X	R54X - Senility	Exclusion
R630	R630 - Anorexia	Exclusion
R633	R633 - Feeding difficulties and mismanagement	Exclusion
T855	T855 - Mechanical complication of gastrointestinal prosthetic devices, implants and grafts	Exclusion
T860	T860 - Bone-marrow transplant rejection	Exclusion
T864	T864 - Liver transplant failure and rejection	Exclusion
Z431	Z431 - Attention to gastrostomy	Exclusion
Z432	Z432 - Attention to ileostomy	Exclusion

ICD10 Code	Description	Category
E244	E244 - Alcohol-induced pseudo-Cushing's syndrome	Alcohol
E512	E512 - Wernicke's encephalopathy	Alcohol
F100	F100 - Mental and behavioural disorders due to use of alcohol. Acute intoxication	Alcohol
F101	F101 - Mental and behavioural disorders due to use of alcohol. Harmful use	Alcohol
F102	F102 - Mental and behavioural disorders due to use of alcohol. Dependence syndrome	Alcohol
F103	F103 - Mental and behavioural disorders due to use of alcohol. Withdrawal state	Alcohol
F104	F104 - Mental and behavioural disorders due to use of alcohol. Withdrawal state with delirium	Alcohol
F105	F105 - Mental and behavioural disorders due to use of alcohol. Psychotic disorder	Alcohol
F106	F106 - Mental and behavioural disorders due to use of alcohol. Amnesic syndrome	Alcohol
F107	F107 - Mental and behavioural disorders due to use of alcohol. Residual and late-onset psychotic disorder	Alcohol
F108	F108 - Mental and behavioural disorders due to use of alcohol. Other mental and behavioural disorders	Alcohol
F109	F109 - Mental and behavioural disorders due to use of alcohol. Unspecified mental and behavioural disorder	Alcohol
G312	G312 - Degeneration of nervous system due to alcohol	Alcohol
G621	G621 - Alcoholic polyneuropathy	Alcohol
G721	G721 - Alcoholic myopathy	Alcohol
I426	I426 - Alcoholic cardiomyopathy	Alcohol
K292	K292 - Alcoholic gastritis	Alcohol
K700	K700 - Alcoholic fatty liver	Alcohol
K702	K702 - Alcoholic fibrosis and sclerosis of liver	Alcohol
K746	K746 - Other and unspecified cirrhosis of liver	Alcohol
K852	K852 - Alcohol-induced acute pancreatitis	Alcohol
K860	K860 - Alcohol-induced chronic pancreatitis	Alcohol
X450	X450 - Accidental poisoning by and exposure to alcohol. Home	Alcohol
X451	X451 - Accidental poisoning by and exposure to alcohol. Residential institution	Alcohol
X452	X452 - Accidental poisoning by and exposure to alcohol. School, other institution and public administrative area	Alcohol
X453	X453 - Accidental poisoning by and exposure to alcohol. Sports and athletics area	Alcohol
X454	X454 - Accidental poisoning by and exposure to alcohol. Street and highway	Alcohol
X455	X455 - Accidental poisoning by and exposure to alcohol. Trade and service area	Alcohol
X456	X456 - Accidental poisoning by and exposure to alcohol. Industrial and construction area	Alcohol

ICD10 Code	Description	Category
X457	X457 - Accidental poisoning by and exposure to alcohol. Farm	Alcohol
X458	X458 - Accidental poisoning by and exposure to alcohol. Other specified places	Alcohol
X459	X459 - Accidental poisoning by and exposure to alcohol. Unspecified place	Alcohol
X650	X650 - Intentional self-poisoning by and exposure to alcohol. Home	Alcohol
X651	X651 - Intentional self-poisoning by and exposure to alcohol. Residential institution	Alcohol
X652	X652 - Intentional self-poisoning by and exposure to alcohol. School, other institution and public administrative area	Alcohol
X653	X653 - Intentional self-poisoning by and exposure to alcohol. Sports and athletics area	Alcohol
X654	X654 - Intentional self-poisoning by and exposure to alcohol. Street and highway	Alcohol
X655	X655 - Intentional self-poisoning by and exposure to alcohol. Trade and service area	Alcohol
X656	X656 - Intentional self-poisoning by and exposure to alcohol. Industrial and construction area	Alcohol
X657	X657 - Intentional self-poisoning by and exposure to alcohol. Farm	Alcohol
X658	X658 - Intentional self-poisoning by and exposure to alcohol. Other specified places	Alcohol
X659	X659 - Intentional self-poisoning by and exposure to alcohol. Unspecified place	Alcohol
Y150	Y150 - Poisoning by and exposure to alcohol, undetermined intent. Home	Alcohol
Y151	Y151 - Poisoning by and exposure to alcohol, undetermined intent. Residential institution	Alcohol
Y152	Y152 - Poisoning by and exposure to alcohol, undetermined intent. School, other institution and public administrative area	Alcohol
Y153	Y153 - Poisoning by and exposure to alcohol, undetermined intent. Sports and athletics area	Alcohol
Y154	Y154 - Poisoning by and exposure to alcohol, undetermined intent. Street and highway	Alcohol
Y155	Y155 - Poisoning by and exposure to alcohol, undetermined intent. Trade and service area	Alcohol
Y156	Y156 - Poisoning by and exposure to alcohol, undetermined intent. Industrial and construction area	Alcohol
Y157	Y157 - Poisoning by and exposure to alcohol, undetermined intent. Farm	Alcohol
Y158	Y158 - Poisoning by and exposure to alcohol, undetermined intent. Other specified places	Alcohol
Y159	Y159 - Poisoning by and exposure to alcohol, undetermined intent. Unspecified place	Alcohol
Y573	Y573 - Alcohol deterrents	Alcohol
Y901	Y901 - Blood alcohol level of 20-39 mg/100 ml	Alcohol
Y902	Y902 - Blood alcohol level of 40-59 mg/100 ml	Alcohol
Y903	Y903 - Blood alcohol level of 60-79 mg/100 ml	Alcohol
Y904	Y904 - Blood alcohol level of 80-99 mg/100 ml	Alcohol
Y905	Y905 - Blood alcohol level of 100-119 mg/100 ml	Alcohol

ICD10 Code	Description	Category
Y906	Y906 - Blood alcohol level of 120-199 mg/100 ml	Alcohol
Y907	Y907 - Blood alcohol level of 200-239 mg/100 ml	Alcohol
Y908	Y908 - Blood alcohol level of 240 mg/100 ml or more	Alcohol
Y909	Y909 - Presence of alcohol in blood, level not specified	Alcohol
Y910	Y910 - Mild alcohol intoxication	Alcohol
Y911	Y911 - Moderate alcohol intoxication	Alcohol
Y912	Y912 - Severe alcohol intoxication	Alcohol
Y913	Y913 - Very severe alcohol intoxication	Alcohol
Y919	Y919 - Alcohol involvement, not otherwise specified	Alcohol
Z040	Z040 - Blood-alcohol and blood-drug test	Alcohol
Z721	Z721 - Alcohol use	Alcohol

Supplementary Material 3

Table: Descriptive characteristics

Variable	Value	Number of admissions	Percentage
Age group	1-17	5,709	3%
	18-34	27,043	15%
	35-49	30,945	17%
	50-64	35,696	20%
	65-79	43,783	24%
	80+	35,089	19%
	NA	4,108	2%
Sex	Male	61,898	34%
	Female	120,418	66%
Ethnic group	Asian or Asian British	15,720	9%
	Black or Black British	3,148	2%
	Mixed	1,188	1%
	Not given	1,085	1%
	Not known	4,827	3%
	Not stated	15,516	9%
	Other ethnic group	2,900	2%
	White	137,949	76%

Supplementary Material 4

Table: Admissions for malnutrition with an alcohol-related diagnosis

Financial year	Admissions with alcohol-related diagnosis
01/02	158
02/03	138
03/04	123
04/05	108
05/06	153
06/07	136
07/08	153
08/09	165
09/10	161
10/11	260
11/12	245
12/13	331
13/14	346
14/15	389
15/16	739
16/17	638
17/18	766
18/19	814
19/20	826
20/21	793

Supplementary Material 5

Figure: Selection of admissions with dietary related malnutrition

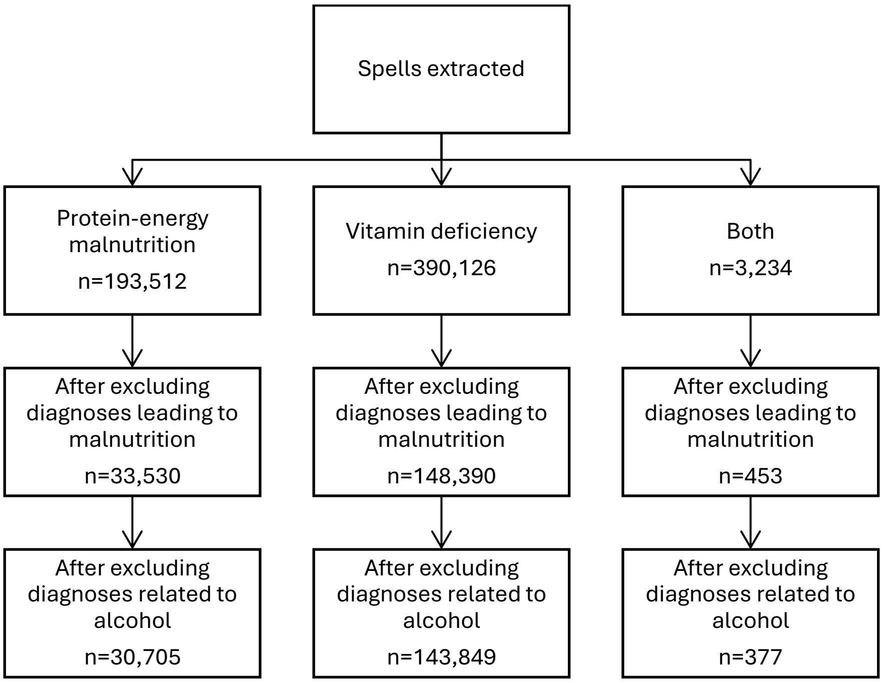

Supplementary Material 6

Figure: Relative risk by middle-layer super output area in England – 2020/21

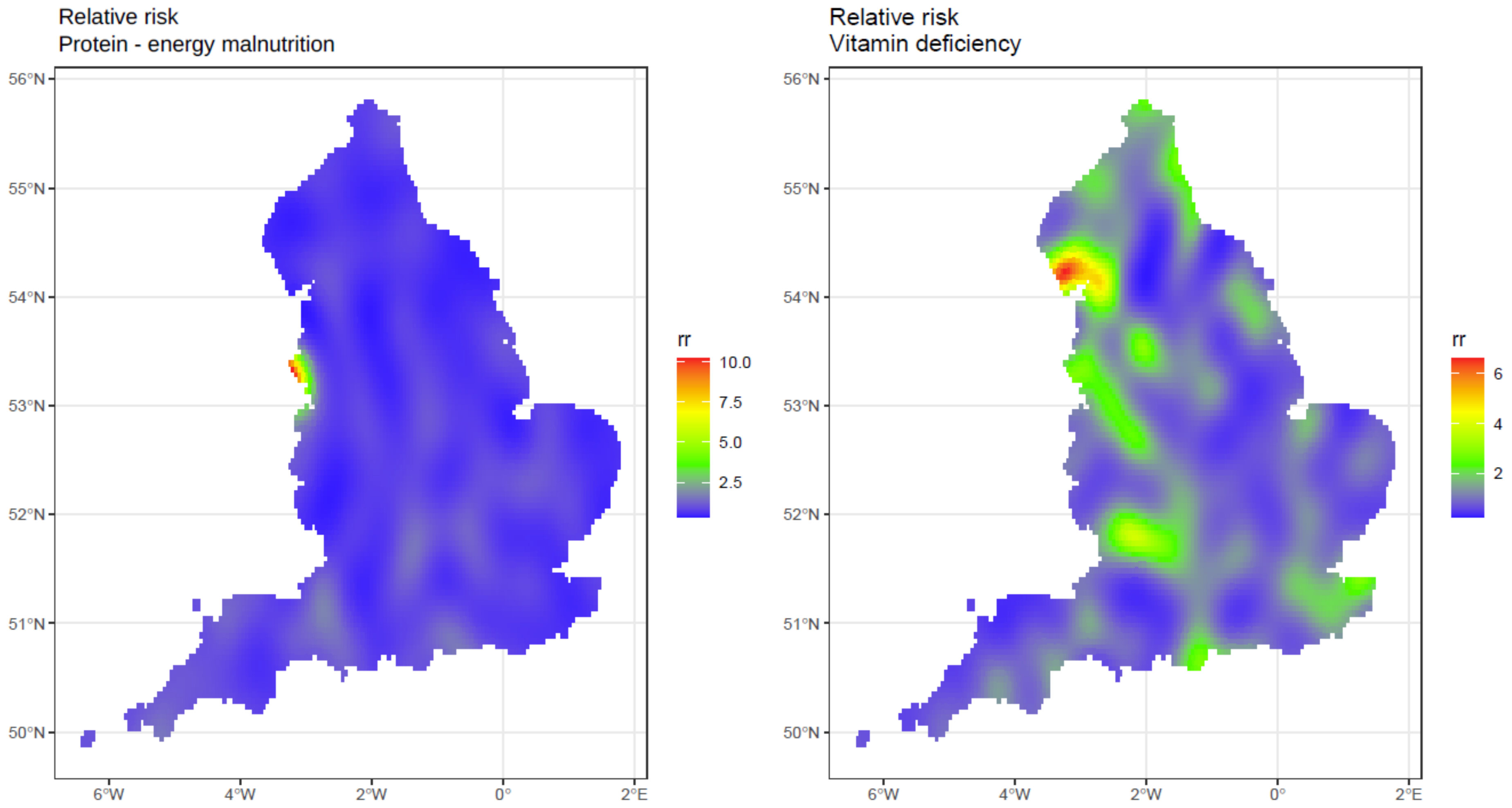

Supplementary Material 7

Figure: Predicted incidence rate ratio for incidence in 2020/21 versus 2010/11 by middle-layer super output area in England

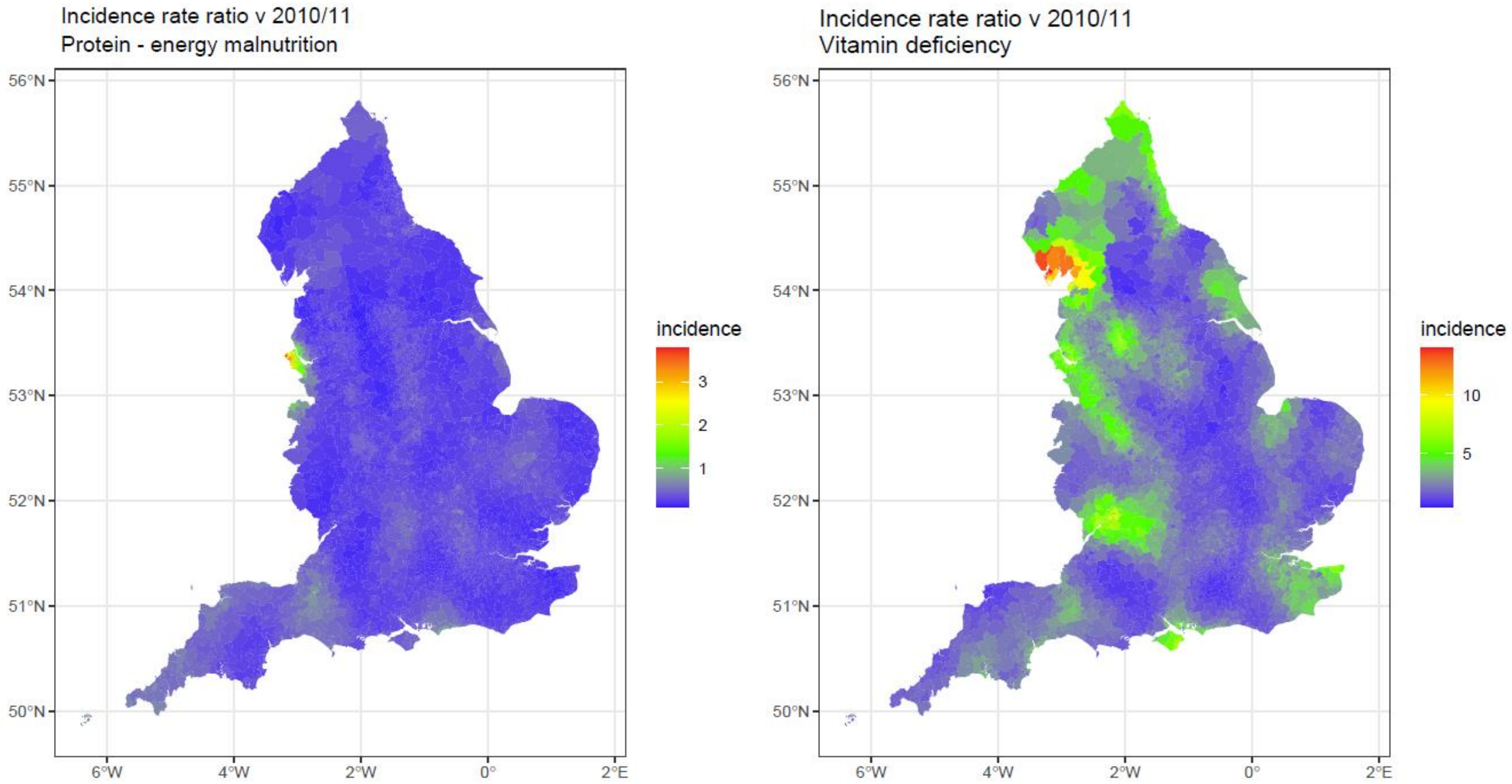

Supplement: online supplemental file 1 [file bmjph-3-2-s001.pdf]
